# *Borrelia burgdorferi* infection modifies protein content in saliva of *Ixodes scapularis* nymphs

**DOI:** 10.1186/s12864-021-07429-0

**Published:** 2021-03-04

**Authors:** Tae Kwon Kim, Lucas Tirloni, Emily Bencosme-Cuevas, Tae Heung Kim, Jolene K. Diedrich, John R. Yates, Albert Mulenga

**Affiliations:** 1 Department of Veterinary Pathobiology, College of Veterinary Medicine, Texas A&M University, College Station, Texas United States of America; 2 Department of Diagnostic Medicine and Veterinary Pathobiology, College of Veterinary Medicine, Kansas State University, Manhattan, Kansas United States of America; 3 Laboratory of Bacteriology, National Institute of Allergy and Infectious Diseases, Hamilton, Montana United States of America; 4 Department of Molecular Medicine, The Scripps Research Institute, La Jolla, California United States of America; 5 Mass Spectrometry Core, Salk Institute for Biological Studies, La Jolla, California United States of America

## Abstract

**Background:**

Lyme disease (LD) caused by *Borrelia burgdorferi* is the most prevalent tick-borne disease. There is evidence that vaccines based on tick proteins that promote tick transmission of *B. burgdorferi* could prevent LD*.* As *Ixodes scapularis* nymph tick bites are responsible for most LD cases, this study sought to identify nymph tick saliva proteins associated with *B. burgdorferi* transmission using LC-MS/MS. Tick saliva was collected using a non-invasive method of stimulating ticks (uninfected and infected: unfed, and every 12 h during feeding through 72 h, and fully-fed) to salivate into 2% pilocarpine-PBS for protein identification using LC-MS/MS.

**Results:**

We identified a combined 747 tick saliva proteins of uninfected and *B. burgdorferi* infected ticks that were classified into 25 functional categories: housekeeping-like (48%), unknown function (18%), protease inhibitors (9%), immune-related (6%), proteases (8%), extracellular matrix (7%), and small categories that account for <5% each. Notably, *B. burgdorferi* infected ticks secreted high number of saliva proteins (*n*=645) than uninfected ticks (*n*=376). Counter-intuitively, antimicrobial peptides, which function to block bacterial infection at tick feeding site were suppressed 23-85 folds in *B. burgdorferi* infected ticks. Similar to glycolysis enzymes being enhanced in mammalian cells exposed to *B. burgdorferi* : eight of the 10-glycolysis pathway enzymes were secreted at high abundance by *B. burgdorferi* infected ticks. Of significance, rabbits exposed to *B. burgdorferi* infected ticks acquired potent immunity that caused 40-60% mortality of *B. burgdorferi* infected ticks during the second infestation compared to 15-28% for the uninfected. This might be explained by ELISA data that show that high expression levels of immunogenic proteins in *B. burgdorferi* infected ticks.

**Conclusion:**

Data here suggest that *B. burgdorferi* infection modified protein content in tick saliva to promote its survival at the tick feeding site. For instance, enzymes; copper/zinc superoxide dismutase that led to production of H_2_O_2_ that is toxic to *B. burgdorferi* were suppressed, while, catalase and thioredoxin that neutralize H_2_O_2_, and pyruvate kinase which yields pyruvate that protects *Bb* from H_2_O_2_ killing were enhanced. We conclude data here is an important resource for discovery of effective antigens for a vaccine to prevent LD.

**Supplementary Information:**

The online version contains supplementary material available at 10.1186/s12864-021-07429-0.

## Background

Tick-borne diseases account for the largest share of human vector-borne diseases reported in the US and its territories. Between 2004 and 2016, nearly 650,000 cases of human vector-borne diseases were reported in the US and its territories, of which 75% (491,671) were tick-borne. During this period, Lyme disease (LD) caused by *Borrelia burgdorferi* (*Bb*), accounted for 82% (402,502/491,671) of the reported human TBD cases [1]. Currently, vaccines against major human tick-borne disease including LD are not available and the primary prevention efforts rely on avoidance of infectious tick bites through different methods which include personal protection equipment, reduction of infected tick populations by means of landscape management, and application of acaricides to kill vector ticks that infest wildlife blood meal sources [1–7]. Despite the availability of these tick control measures, annual LD cases of nearly 30,000 are reported to the US Centers for Disease Control, with actual cases estimated to exceed 300,000 annually [1]. This has justified the need to develop alternative LD prevention methods, and tick-antigen based vaccines have emerged as the most promising [8–10]. The feasibility of using vaccines to control tick infestation was validated with the commercialization the vaccine against cattle fever ticks, *Rhipicephalus* (*Boophilus*) *microplus* that was based on a Bm86 tick midgut antigen [11]. The major limitation has been the lack of effective target antigens to control geographically distinct and other tick species.

There is evidence that tick saliva proteins facilitate transmission of *B. burgdorferi* and other tick-borne disease agents [12]. Importantly the concept of tick-antigen based vaccines as a strategy to prevent LD and other tick-borne disease infections is based on findings that the repeated inoculation of tick saliva proteins into repeatedly infested animals provoked significant anti-tick immunity that resulted in ticks failing to feed and transmitting *B. burgdorferi* [13, 14]. These findings have resulted in a renewed interest in targeting tick saliva proteins in vaccines to prevent LD and other tick-borne disease infections [9, 10, 15]. The approach toward developing tick salivary antigen-based vaccines to prevent tick-borne disease infections has been to understand the molecular basis of tick feeding physiology as a means through which, we can discover effective tick saliva proteins for tick-antigen based vaccines. The starting point of this research is the discovery and characterization of proteins in saliva of ticks that regulate tick feeding and facilitate in the transmission of tick-borne disease agents, which has been attempted through transcriptomics [16–18], immuno-proteomics [19, 20], and tick saliva proteomics [21–26]. There is evidence that tick-borne disease infections of ticks modified transcription and protein expression patterns in different tick species [27–31]. Prompted by these findings, the objective of this study was to identify proteins in saliva of *B. burgdorferi-*infected *I. scapularis* nymphs every 12 h of feeding. Although adult ticks are capable of transmitting *B. burgdorferi,* we focused on nymphs since majority of human LD infections reported was associated with infectious nymph tick bites [32–34].

Here we used LC-MS/MS to identify and bioinformatically characterize 747 proteins in saliva of uninfected and *B. burgdorferi-*infected *I. scapularis* nymphs at different stages of the tick feeding process: unfed, those that partially-fed on rabbits for 12, 24, 36, 48, 60, and 72 h, and replete fed. Our data show that *B. burgdorferi* infection modified the protein content in saliva of infected *I. scapularis* nymph ticks. The approach described here has allowed us to identify *I. scapularis* nymph tick saliva proteins that are suppressed, induced, enhanced, or not affected in response to *B. burgdorferi* infection of *I. scapularis* nymphs. Of particular significance, saliva of *B. burgdorferi* infected ticks contained higher number of proteins than the uninfected. We speculate that this could explain our observation that a single infestation of rabbits by *B. burgdorferi* infected ticks provoked strong anti-tick immunity that resulted in 40-60% mortality of *B. burgdorferi* infected ticks during the second infestation compared to 20% on uninfected rabbits. We discuss our data with reference to understanding the molecular basis of tick transmission of the LD agent**.**

## Results

### Exposing *Ixodes scapularis* nymph tick mouthparts to pilocarpine induces salivation

*Borrelia burgdorferi* infected *I. scapularis* nymphs that were used in this study were successfully infected by artificially feeding [35] and summarized in supplemental figure (SF) 1. Our rationale to artificially infect ticks was to ensure 100% *B. burgdorferi* infection in all ticks that were used for saliva collection. We confirmed that *B. burgdorferi* infected nymph ticks were able to transmit *B. burgdorferi* to rabbits by ELISA and western blotting analyses of cultured *B. burgdorferi* protein extracts using purified IgG of rabbits that were infested by uninfected and *B. burgdorferi* infected ticks (SF2A and SF2B).

Prior to this study, collection of tick saliva required injecting ticks with pilocarpine or applying pilocarpine-ethanol solution on the dorsal side of the tick to stimulate salivation and then collect saliva into capillary tubes that were affixed onto tick mouthparts [36]. Given the smaller size of *I. scapularis* nymphs injecting pilocarpine was problematic, as majority of nymphs could not survive the injection trauma. In this study, we have validated a non-invasive approach to collect tick saliva by allowing nymph ticks to salivate directly into pipette tips that contained 2% pilocarpine in PBS pH 7.4 (as shown in SF1). We successfully collected sufficient amount of nymph tick saliva by inserting *I. scapularis* nymph tick mouthparts into 10 μL pipette tips that contained 2% pilocarpine solution (5 μL). This allowed ticks to salivate directly into pilocarpine and prevented saliva from drying out. We successfully collected ~13, 9, 20, 18, 37, 27, 43, and 20 μg total saliva proteins from unfed (*n*=50), partially-fed on rabbits for 12, 24, 36, 48, 60, and 72 h (*n*=30 per time point) and replete-fed (*n*=15) *B. burgdorferi* infected ticks. Likewise, we collected 11, 18, 18, 13, 21, 11, 15, and 9 μg of uninfected tick saliva proteins respectively. To validate and visualize protein profiles in collected saliva, ~1.5 μg of total tick saliva proteins per time point were resolved on SDS-PAGE and analyzed by silver staining (SF3).

### *Borrelia burgdorferi*-infected *Ixodes scapularis* nymphs secrete more proteins than the uninfected

To identify proteins in tick saliva, 2 μg of total tick saliva protein per run (in triplicate) was analyzed by LC-MS/MS as described [21–26]. A notable finding in this study is that *B. burgdorferi* infected ticks secreted high number of saliva proteins (*n*=645) compared to the uninfected ticks (*n*=376). Overall, we identified a combined 747 tick proteins in saliva of uninfected and *B. burgdorferi-*infected *I. scapu4laris* nymphs that were classified in 25 functional categories (Supplemental Table (ST) 1 and 2). Except for replete fed (96-120 h fed) *B. burgdorferi* infected ticks that secreted fewer proteins compared to uninfected (2.2 folds lower), all other *B. burgdorferi* infected feeding stages secreted more proteins than uninfected by factors of 4.3 folds in unfed ticks and 1.7, 2.2, 1.9,1.5, 2.1 and 2.6 folds in 12, 24, 36, 48, 60, and 72 h fed ticks respectively (Fig. . 1 and ST2).

**Fig. 1:**
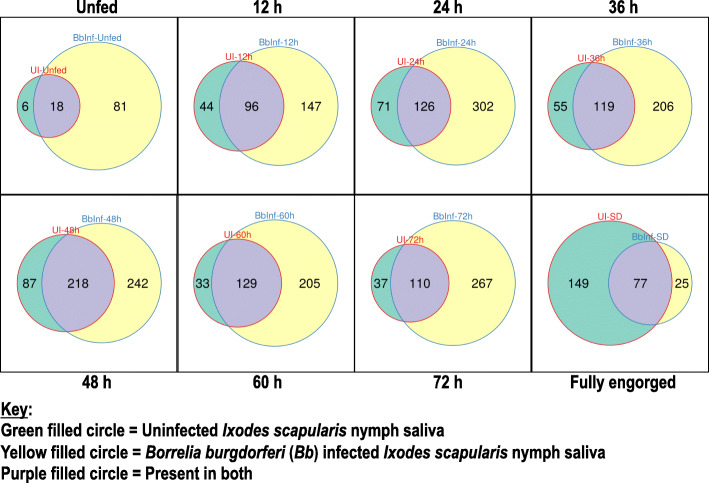
*Borrelia burgdorferi* infection of *Ixodes scapularis* nymphs induces secretion of a high number of tick saliva proteins. Total numbers of tick saliva proteins identified at each tick feeding time point (unfed, 12h, 24h, 36h, 48h, 60h, 72h, and fully engorged [SD]) were enumerated. Green, Yellow, and Purple circles = the number of tick saliva proteins respectively identified in uninfected (UI), *Borrelia burgdorferi* infected (Bbinf), and found in both

Antimicrobial peptides (AMP) in tick saliva are expected to suppress bacteria growth at the tick feeding site and clear microbes in blood meal [37]. Therefore, it is noteworthy that *B. burgdorferi* infection completely blocked secretion of 4 of the 5 AMPs that were identified in uninfected ticks (ST1). In contrast, two functional categories: amino acid metabolism and transcription machinery proteins were only detectable in saliva of *B. burgdorferi* infected ticks (ST2); suggesting that secretion of these proteins into tick saliva was induced by *B. burgdorferi* infection. With exception of three functional categories (nuclear regulation, glycine rich, and mucin) for which both uninfected and *B. burgdorferi* infected ticks secreted the same number of proteins, *B. burgdorferi* infection caused ticks to secrete more proteins for the remaining functional categories. *B. burgdorferi* infection caused ticks to secrete more proteins by factors of between 2.0-16 folds in functional categories of metabolism proteins (carbohydrates, nucleic acids, energy and lipids), immune related, proteases, transporters/ receptors, signal transduction, protein export, and proteasome machinery. Likewise, saliva of *B. burgdorferi* infected ticks contained more proteins by factors between 1.1-1.9 folds for protease inhibitors, tick-specific proteins of unknown function, detoxification/antioxidant, cytoskeletal, extracellular matrix, protein modification, heme/ iron binding, protein synthesis, and histamine-binding proteins/ lipocalins (ST2).

### *Borrelia burgdorferi* infection modifies secretion dynamics of *Ixodes scapularis* nymphs tick saliva proteins

**Fig. 2:**
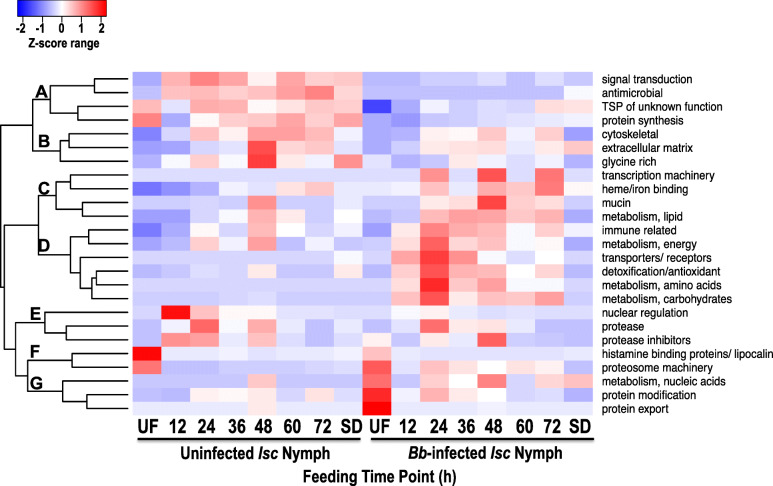
*Borrelia burgdorferi* (*Bb*) infection modifies protein content on composition of *Ixodes scapularis* nymphs. Cumulative normalized spectral abundance factor (NSAF) value, the index for relative protein abundance for all proteins in saliva of uninfected and *Bb* infected nymph ticks was normalized using the z-score statistics and then used to generate heat maps using heatmap2 function in gplots library using R as described in materials and methods. The red to blue transition denotes high to low abundance levels shown in the Z-score range key. The reader is advised that the raw NSAF values that were used to generate the heatmap are provided in S1 Table

To gain further insight into temporal secretion dynamics of proteins at different tick feeding timepoints, the sum total of the normalized spectral abundance factor (NSAF), an index for relative protein abundance [38–41] for each functional category was used to generate the heatmap using R package [41] (Fig. 2; NSAF values used to generate heatmaps shown in ST2). The heatmap reveals seven secretion pattern trends: branches A-G. Overall the sum total relative abundance of tick saliva proteins in branch A (signal transduction, antimicrobial, TSP of unknown function, and protein synthesis), branch B (cytoskeletal, extracellular matrix, and glycine rich), nuclear regulation in branch E, lipocalin in branch F were suppressed in saliva of *B. burgdorferi* infected ticks. Likewise, functional categories in branch B (cytoskeletal, extracellular matrix, and glycine rich) and branch E (protease and protease inhibitors) were suppressed in response to *B. burgdorferi* infection. In contrast, tick saliva proteins in 14 functional categories were secreted at high abundance by *B. burgdorferi* infected ticks. These include transcriptional machinery, heme/ion binding, mucin, and lipid metabolism in branch C; immune related, energy metabolism, transporters/receptors, detoxification/antioxidant, amino acid metabolism, and carbohydrate metabolism in branch D; and proteasome machinery, nucleic acid metabolism, protein modification, and protein export in branch G. Finally, proteases and protease inhibitors in branch E were secreted by both uninfected and infected ticks at comparable levels. Overall, data in Fig. 2 provides insights into functional categories that might be associated with transmission of *B. burgdorferi* as these were secreted at high abundance by *B. burgdorferi* infected ticks.

### *Borrelia burgdorferi* infection modifies relative abundance of *Ixodes scapularis* nymph tick saliva proteins

**Fig. 3:**
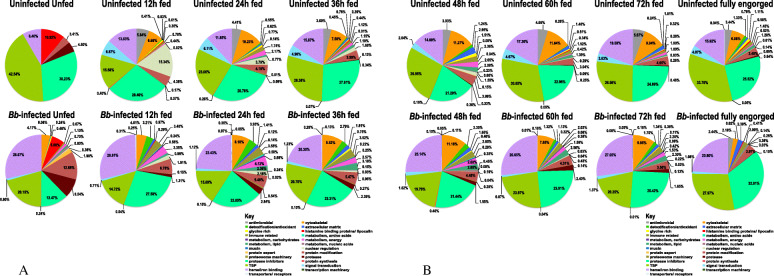
Temporal relative abundance of proteins in saliva of uninfected and *Borrelia burgdorferi* (*Bb*) infected *Ixodes scapularis* nymphs. Relative abundance (based on average normalized spectral abundance factors) for each functional category is expressed as a percent (%) of total protein content of unfed and 12, 24, and 36 h fed ticks (**a**) and 48, 60, 72h, and replete fed ticks (**b**). Please read the key from left to right and the pie chart in a clockwise direction, starting from the antimicrobial peptides (grey color). Please note that antimicrobial peptides are absent in unfed ticks, and are less than 1% in *Bb* infected ticks

To gain further insight on the effects of *B. burgdorferi* infection on relative abundance tick saliva protein functional categories, sum totals of NSAF values for each functional category, the index for relative protein abundance [38–41] were expressed as percent of relative total protein content per time point (Fig. 3). The most abundant categories in both uninfected and *B. burgdorferi-*infected ticks belong to protease inhibitors (chartreuse green), TSPs (green), heme/iron binding proteins (purple), cytoskeletal (orange), proteases (brown), and antimicrobial (gray); with the rest of categories accounting for less than 4% each. Fold changes in relative protein abundance between uninfected and *B. burgdorferi* infected ticks are summarized in ST2. The most notable effects of *B. burgdorferi* infection is on relative abundance of AMPs (gray) in tick saliva, which was suppressed by 23-85 folds: reducing from 3-6% of total proteins in uninfected tick saliva to less than 1% in *B. burgdorferi* infected ticks during the 12-72 h feeding period, but not in replete fed (96-120 h fed) ticks where relative abundance is apparently similar. As shown in ST2, the other functional categories that were suppressed in response to *B. burgdorferi* infection include lipocalin (1.2-2.5 folds), TSP of unknown function (1.2-2.1 folds), signal transduction (1.6-9.6 folds), nuclear regulation (1.7-8.0 folds), and glycine rich proteins (0.6-6.6 folds). The effects of *B. burgdorferi* infection on protease inhibitors was mixed in that, relative abundance was reduced by 1-2.4 folds in unfed and 12-36 h fed ticks: however relative abundance was increased by up to 1.28 folds in 48-72 h fed and replete fed (96-120 h fed) ticks. Similarly, the protein synthesis functional category was suppressed by up to 1.2-11 folds for 12, 24, and 60 h fed ticks, but enhanced by 0.8-2.4 folds in unfed and 36, 48, and 72 h fed ticks. The same pattern was observed for cytoskeletal proteins; suppressed by 1-2.8 folds in 12, 24, 48, 60 h fed and replete fed (96-120 h fed), but enhanced by 1-4.2 folds in unfed, 36 and 72 h fed ticks.

### Secretion dynamics profiling reveals proteins likely associated with transmission of *Borrelia burgdorferi*

The heatmap in SF4 highlights the overall secretion dynamics of all 747 tick saliva proteins based on NSAF values (present in at least 2 of the 3 runs per time point, raw values in ST1) as index for relative abundance. Similar to observations in Fig. 2, the heatmap for all proteins in SF4 reveals that *B. burgdorferi* infection had three broad effects: suppression, enhancement or unmodified (no effect) on tick saliva secretion.

**Fig. 4:**
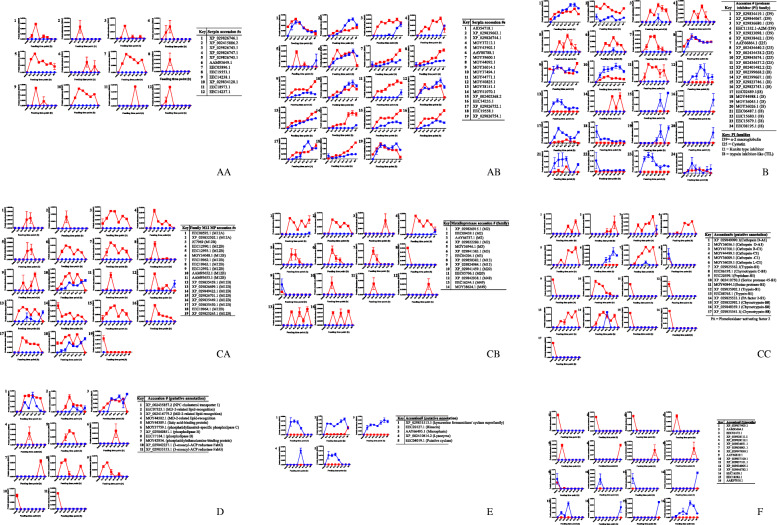
Time resolved secretion dynamics of proteins in saliva of uninfected and *Borrelia burgdorferi (Bb)* infected. The normalized spectral abundance factor as the index for relative protein abundance was graphed using PRISM 8 for five functional protein categories of uninfected (blue line) and Bb infected (red line) tick saliva during feeding. (AA) represent serine protease inhibitors (serpin) secreted by *Bb* infected saliva only, (AB) represent serpins secreted by both uninfected and *Bb* infected ticks, (B) = non-serpin protease inhibitors; (CA) represent M12 proteases, (CB) represent other metalloproteases, and (CC) represent other proteases, (D) represent cholesterol and lipid binding proteins, (E) represent antimicrobial peptides, and (F) represent histamine binding proteins/ lipocalins. Please note that the table insert that is included in the figure lists accession numbers and putative annotations. The X-axis data points represent unfed stage, feeding time points every 12 h through 72 h, and replete-fed ticks. The Y-axis represents relative abundance as indicated by average NSAF values from the three LC-MS/MS runs

To gain insight on effects of *B. burgdorferi* infection on individual proteins in this study, a selected group of functional categories (protease inhibitors, proteases, lipid metabolism-associated proteins, antimicrobial peptides, and histamine binding proteins/ lipocalins) were graphed in PRISM8 (Graphpad Software Inc., San Diego CA, USA) (Figs. 4a-f). Secretion dynamics of the remaining functional categories are presented as heatmaps in SF5. Tick housekeeping protein-like functional categories have been discussed as a group, with commentary on key highlights. We have also commented on the effect of *B. burgdorferi* infection on secretion of rabbit proteins in tick saliva. To provide context, we have commented on probable functional roles of tick saliva proteins tick transmission of *B. burgdorferi.*

#### Protease inhibitors (PI)

We previously showed that ticks encoded at least 18 of the 79 protease inhibitors (PI) families that are listed in the MEROPS database [42–44]. In this study, we identified 71 PIs in five PI families: Kunitz type (I2, n=1), serine protease inhibitors (serpin, family I4, n=34), trypsin inhibitor-like (TIL, family I8, n= 17), cystatin (family I25, n=10), and α-2 macroglobulin (family I39, A2M, n=9) (Figs. 4AA, 4AB, and 4B: Due to high of PIs, we have split Fig. 4A into 4AA and 4AB [serpins] and 4B [non-serpin protease inhibitors]). Similar to protein content in saliva of adult ticks [22, 24, 26], data here show that majority of the PIs in saliva of both uninfected and *Bb* infected nymphs were serine protease inhibitors (serpins). Although serpins are the majority PIs in tick saliva, they are second behind family I2 (Kunitz serine PI) among tick-encoded PIs [43]. Interestingly, we found a single family I2 PIs in nymph saliva, and they accounted for only 2 of the 43 PIs that were identified in saliva of adult *I. scapularis* [24]. These findings might suggest a limited role for Kunitz-type serine protease inhibitors in regulating feeding and transmission of *B. burgdorferi* by ticks.

##### Serpins

The tick feeding style of disrupting host tissue and then imbibing the host blood that bleeds into the wounded lesion is expected to trigger host defense pathways that initiate wound healing and prevent further blood loss. The host defense pathways triggered by tick feeding such as hemostasis, complement, and inflammation pathways are mediated by serine proteases that are under tight regulation by serpins [45–48]. From this perspective, it has been hypothesized that ticks might utilize serpins to evade host defense [49–51]. On this basis it is notable that 34 of the 71 PIs found in this study are serpins. Due to the high number of serpins, we have split them into those identified in saliva of *B. burgdorferi* infected ticks only (Fig. 4AA, *n*=13) and those identified in saliva of both uninfected and *B. burgdorferi* infected ticks (Fig. 4AB, *n*=21). We would like the reader to note here and elsewhere that representative graphs are shown for serpins (and other proteins) that display similar secretion patterns. Serpins showing similar secretion patterns include Fig. 4AA (4XP_029826747.1 and EEC19554.1), Fig. 4AB2 (XP_029839663.1 and EEC08061.1), and Fig. 4AB8 (MOY44092.1 and XP_029826753.1). It is notable that the relative abundance for majority of serpins that were secreted by *B. burgdorferi* infected tick only was highest at 24-48 h tick feeding time points (Fig. 4AA) preceding the major transmission events of *B. burgdorferi* after the tick has fed for more than 48 h [32–34]. It is also notable that 12 of the 21 serpins that were secreted by both uninfected and *B. burgdorferi* infected ticks were enhanced in *B. burgdorferi* response to infection (Fig. 4AB) suggesting the possibility that they are associated with transmission of *B. burgdorferi*.

Some of the serpins in this study were previously characterized including AID54718.1 (Fig. 4AB1) as an inhibitor of thrombin, blood clotting and platelet aggregation [52]. Similarly, EEC18973.1 (Fig. 4AA11), is one of the highest conserved tick serpins known to date; its functional reactive center loop is 100% conserved in tick species where data was available [52, 53]. Importantly, RNAi silencing of EEC18973.1 [54] or its homolog in *A. americanum* (AAS19) [55] caused deformities in ticks, reduced blood meal sizes, and tick mortalities. Indirect evidence based on functional analysis of AAS19 suggests that EEC18973.1 is likely a glycosaminoglycan-binding serpin and an inhibitor of platelet aggregation and blood clotting [53, 56]. It is also notable that, XP_002402368.2 (Fig. 4AB15) is a homolog to *I. ricinus* ABI94056.2, an anti-inflammatory protein that acts by inhibiting chymase and cathepsin G [57]. Taken together, the abundance of these serpins in *B. burgdorferi* infected ticks at early stages of feeding could be important in responding to early host defense such as hemostasis and inflammation for successful transmission and dissemination of *B. burgdorferi* into the mammalian host.

##### Non-serpin protease inhibitors

The 37 non-serpin PIs include alpha-2 macroglobulin (*n*=9; A2M, Fig. 4B1-6, note that four A2M, XP_029834419.1, XP_029834431.1, XP_029834426.1, and MOY43687 are represented by Fig. 4B1), ten cysteine protease inhibitors (cystatins, Fig. 4B7-11, AAY66864.1 and MOY41952.1, XP_002434440.2 and Q8MVB6.1, XP_029845674.1, B7PKZ1.1 and 3MWZ.1 are respectively represented by Fig. 4B7, Fig. 4B8; and Fig. 4B10). The other non-serpin PIs include a single Kunitz type inhibitor (Fig. 4B12), and 17 trypsin inhibitor-like inhibitors (TIL, Fig. 4B13-24) with XP_002399668.2 and XP_029825278.1; XP_029823746.1 and MOY36044.1; XP_029823743.1, MOY43102.1 and MOY43175.1; and .1 and XP_029823423.1 are respectively represented in Fig. 4B13, Fig. 4B15, Fig. 4B16, and Fig. 4B17).

Except for the A2M in Fig. 4B1 that was not affected by *B. burgdorferi* infection, the rest of A2Ms were secreted at high abundance in saliva of *B. burgdorferi* infected ticks (Figs. 4B2-6). In mammals, A2M are known for their roles as broad-spectrum inhibitors of proteases and regulators of immune factors such as cytokine, IL-1, IL-6 and growth factors [58]. Whether or not tick A2Ms retain these functions remains to be investigated. There is limited evidence that tick A2M might regulate tick and pathogen interactions as suggested by up regulation of A2M in *Rickettsia montanensis-*infected *D. variabilis* ovaries indicating roles in transovarial transmission [59] and regulating phagocytosis of tick hemocytes indicates roles in tick immunity [60].

Of the 10 cystatins, two were enhanced (Figs. 4B7 and 11), two were unmodified (Figs. 4AB8 and 9), three were suppressed in *B. burgdorferi* infected ticks (Fig. 4B10), and three were only identified in *B. burgdorferi* infected ticks (S1 Table). There is evidence that cystatins play roles in tick and pathogen interactions [61]. Some of the tick cystatins have inhibitory activities against cysteine proteases and have immunomodulatory functions, which will be beneficial to transmitted *B. burgdorferi* [61]. One surprising finding in our data is that XP_029845674.1 (Fig. 4B10) which is Sialostatin-L2, an inhibitor of cathepsin-L, immuno-suppressant, and enhancer of *B. burgdorferi* proliferation in culture, was suppressed in saliva of *B. burgdorferi* infected ticks. On the other hand, XP_002434440.2 (Fig. 4B8), which is 100% identical to Sialostatin L, also an immuno-suppressant [61] was apparently not modified with *B. burgdorferi* infection.

Except for XP_002399667.1 (Fig. 4B14), TILs (Fig. 4B12, 15-24) were suppressed in *B. burgdorferi* infected tick saliva which suggests that they play a limited role(s) in *B. burgdorferi* transmission. Similarly, the single Kunitz type inhibitor in this study was suppressed in *B. burgdorferi* infected ticks and was present only in uninfected tick saliva throughout feeding (Fig. 4B12).

#### Proteases

Functional roles of proteases have been linked to several aspects of tick physiology [62–66] and transmission of tick-borne disease agents [67]. In this study, we found 58 proteases in saliva of uninfected and *B. burgdorferi* infected *I. scapularis* nymphs (Fig 4c). Consistent with published data in adult *I. scapularis* tick saliva proteome [24], majority of proteases in saliva of *I. scapularis* nymphs are metalloproteases (MPs) (38/58), followed by serine proteases (12/58), cysteine proteases (5/58), and aspartic proteases (3/58) (Fig. 4CA, CB, and CC, S1 Table). The *I. scapularis* genome encodes for at least 150 and 233 putatively inactive and active proteases, with majority being MPs, followed by serine proteases, cysteine proteases, aspartic proteases and threonine proteases [68]. Given that the *I. scapularis* genome encodes for more MPs followed by serine proteases, it is potentially possible that our findings here might be the consequence of the protease composition in *I. scapularis* genome. However, we are of the view that this might not be the case in that secretion of majority of proteases in this study were enhanced in saliva of *B. burgdorferi* infected ticks. Given the high number of protease sequences, we have separated secretion dynamics in three figures for M12B MPs (Fig. 4CA), non-family M12B MPs (Fig. 4CB), and non-MP proteases (Fig. 4CC).

##### Metalloproteases (MPs)

Of the 38 MPs in this study, 21 are in family M12, two of which are in subfamily M12A (Figs. 4CA1 and 2) and the rest are in subfamily M12B (Figs. 4CA3-19). Please note that Fig. 4CA11 represents secretion dynamics of AAM93653.1 and XP_029826700.1, while Fig. 4CA17 represent XP_029835450.1 and XP_029883546.1. Non-family M12 MPs are in families M2 (Figs. 4CB1-4), M3 (Figs. 4CB5-7: please note that Fig. 4CB5 represent MOY36946.1, XP_029839442.1 and EEC16348.1), M13 (Figs. 4CB8 and 9), M20 (Figs. 4CB10 and 11, please note that Fig. 4CB10 represent XP_029841459.1 and MOY43909.1), and M49 (Figs. 4CB12-14).

The finding that majority of MPs in this study are in subfamily M12B that are structurally similar to snake venom MPs is interesting. Functional properties of snake venom MPs include hemorrhaging, degrading extracellular matrices, fibrinolysis, and inhibiting platelet aggregation [69]. Given that functions of snake venom MPs will promote tick feeding and dissemination of transmitted *B. burgdorferi* from the site of inoculation, it is logical that *B. burgdorferi* infected nymphs secreted these proteases at high abundance*.* It is notable that similar to snake venom functions, degradation of gelatin, fibrinogen, and fibronectin were reported in *I. scapularis* saliva [70]. Interestingly, the MP that were isolated from saliva and cloned by Francischetti et al., [70] (AY264367) is 100% identical to JC969 which was enhanced in response to *B. burgdorferi* infection in this study (Fig. 4CA3). Likewise, RNAi-mediated silencing of Metis 1 and Metis 2, which have 89% amino acid residue identity to AAM93652.1 (Fig. 4CA10) resulted in reduced fibrinogenolytic activity of *I. ricinus* saliva [71].

##### Non-metalloproteases proteases

Non-metalloproteases include three aspartic proteases (AP, Figs. 4CC1 and 2, please note that XP_029849090 and MOY36011.1 are represented in Fig. 4CC1), five cysteine proteases (Figs. 4CC3-6; note that Fig. 4CC5 represent MOY36009.1 and MOY36012.1), and 12 serine proteases (Figs. 4CC7-17; note that Fig. 4CC14 represent XP_029825533.1 and XP_029825536.1). It is notable in Fig. 4CC that majority of non-metalloproteases were enhanced in response to infection suggesting the potential for these proteases to play roles in transmission of *B. burgdorferi*.

The 12 serine proteases in this study have features of subfamily S1 proteases that represent the core of mediators of the host defense system against tick feeding such as blood clotting, complement, and inflammation [72–74]. Additionally, S1 proteases were more abundant in *B. burgdorferi* infected ticks, with four of these present only in *B. burgdorferi* infected ticks when compared to the uninfected ticks (Figs. 4CC7-17). From this perspective, it is conceivable that ticks might secrete S1 serine proteases to interfere with the host defense pathway. There is evidence that this might indeed be the case. A trypsin-like S1 serine protease that activates protein-C, was purified from saliva of adult *I. scapularis* [63]*.* Among other functions, activated protein-C regulates coagulation aimed at maintaining the fluidity of blood within the vasculature [75], which will be beneficial to both tick feeding and dissemination of transmitted *B. burgdorferi*. It is also interesting to note that serine protease phenoloxidase−activating factor 2 was secreted by infected ticks during the 12-72 h feeding time points, and only at the 48 h time point in uninfected ticks (Fig. 4CC14). Indirect data in insects [76–78]. suggest that phenoloxidase−activating factor 2 could be involved in the ticks’ immune response to microbial infection. Data in bees also suggest that insect phenoloxidase−activating factor 2 is an activator of prothrombin and degrades fibrinogen to fibrin [79] if functional, the tick protein might interfere with the host blood-clotting defense in this way. Mammalian S1 serine proteases are also critical activators of protease-activated receptors (PARs), which are involved in signaling of host defense pathways [80–82]. that the tick must suppress to successfully feed and transmit tick-borne disease agents. It is conceivable that serine proteases in this study might interact with signaling of PARs to interfere with the host response against tick feeding and host colonization by transmitted *B. burgdorferi.* A notable feature of serine proteases in this study is that majority were secreted highly by *B. burgdorferi* infected unfed ticks, except for five (Figs. 4CC7, 10, 13, and 14) that were secreted throughout the feeding process.

In addition to breaking down of peptides in lysosomes, cysteine proteases play important roles in induction and development of innate and adaptive immunity encompassing antigen-and pathogen-recognition and elimination, signal processing and cell homeostasis [83, 84]. Indirect evidence showing that cysteine proteases regulate key parasite pathogenesis [85–87] suggest the possibility that cysteine proteases identified here could aide transmitted *B. burgdorferi* to evade host immune defenses. It is interesting to note that four of the five cysteine proteases in this study were present in saliva of *B. burgdorferi* infected ticks only (Figs. 4CC3-6). Likewise, functional studies on tick aspartic proteases were limited to roles in physiology such as oocyte maturation and degradation of tick vitellin and host serum albumin [88]. The three aspartic proteases that were identified in this study were present only in *B. burgdorferi* infected tick saliva. Interestingly XP_0298490090.1 (Fig. 4CC1) shows ~50% amino acid identity to an aspartic protease (KC540783.1) of the human itch mite (*Sarcoptes scabei hominis*) which degraded human hemoglobin, serum albumin, fibrinogen and fibronectin [89].

#### Putative cholesterol and other lipid-binding proteins

Figure 4d summarizes the secretion dynamics of putative cholesterol and other lipid binding/interacting proteins. The six putative cholesterol binding proteins that show similarity to mammalian Niemann-Pick type C1 (NPC) (Fig. 4D1: represents XP_002435857.2 and XP_029829620.1) and MD-2-related lipid-recognition (ML) domain proteins (also known as NPC2) (Figs. 4D2-4, MOY44429.1, XP_002414779.2, and EEC18444.1 are represented by Fig. 4D4). The ML domain proteins (NPC2) can bind IgE [90] and microbial lipopolysaccharide (LPS) [91], and thus it will be interesting to investigate if proteins here interact with *B. burgdorferi* products*.* We also would like to note that XP_002435857.2 and XP_029829620.1 (Fig. 4D1) are highly identical except for an 11 amino acid deletion in the C-terminus region (not shown). Likewise, the NPC2 sequences MOY44429.1, XP_002414779.2, and EEC18444.1 (Fig. 4D4) are highly identical with amino acid differences restricted in amino-terminus region.

In mammals, interactions of NPC1 and NPC2 are required in transfer of cholesterol from lysosomes where currently two mechanisms are proposed: (1) NPC2 binds cholesterol which is transferred to NPC1 to be transported into cytosol and to the endoplasmic reticulum (ER), and (2) NPC1 directly binds to cholesterol and transfers it to NPC2 to be transported into the cytosol and to the ER [92]. Whether or not, this is the case for putative tick NPC1 and NPC2 remains to be ascertained. The mammalian NPC1 is ~1300 amino acid residue with 13 transmembrane domains with an amino-terminus cholesterol-binding domain [93]. It is interesting to note that XP_029829620.1 and XP_002435857.1 (Fig 4D1) which are both annotated as NPC1 in GenBank have 274 and 286 amino acid residues, respectively, which is smaller than the human NPC1. It is notable that, the tick NPC1 aligns with the amino-terminus cholesterol-binding domain of the mammalian NPC1 (not shown). Data mining in GenBank revealed that the *I. scapularis* genome encodes for other short NPC1-like proteins (XP_002435856.2, XP_002412172.1, XP_029830588.1 and XP_002402589.2) that were not found in tick saliva. We also would like to note that, *I. scapularis* also encodes for five other putative NPC1 (XP_029824526.1, XP_029824532.1, XP_029824537.1, EEC13272.1, and EEC08477.1) that are more than 1300 amino acid residues long and retain the secondary structure of the mammalian NPC1 but were not found in tick saliva.

The other lipid-binding proteins in Fig. 4d are annotated as putative fatty acid-binding proteins (Fig. 4D5), phosphatidylinositol-specific phospholipase C (Fig. 4D6) that participates in cell signaling through generation of second messengers: inositol 1,4,5-trisphosphate and diacylglycerol [94], and phospholipase B (Fig. 4D7 and 8) that play roles in signal transduction and inflammation through effects on metabolism of phospholipids and lysophospholipids [95]. The remaining lipid binding proteins include phosphatidylethanolamine-binding protein (Fig. 4D9) that is associated with lipid binding and serine protease inhibition [96] and has been shown to have antimicrobial function in *Bombyx mori* [97], and 3-oxoacyl reductase (Fig 4D10 and 11) that is involved in fatty acid elongation [98] and has also been associated fatty acid biosynthesis in *Plasmodium falciparum* [99].

Although it is important to validate function, the finding that *B. burgdorferi* infection enhanced the secretion of cholesterol and other lipid-binding/interacting proteins by *I. scapularis* nymphs could be linked to *B. burgdorferi* transmission and colonization of the host. There is evidence that *B. burgdorferi* acquires cholesterol from the host and incorporates it as cholesterol glycolipids onto its membranes [100, 101]. Based on these data, it is conceivable that the putative cholesterol and other lipid-binding/interacting proteins in this study might bind and present plasma lipids to transmitted *B. burgdorferi* for initial replication and dissemination from site of inoculation.

#### Antimicrobial peptides (AMP)

AMPs in tick saliva function to suppress bacteria growth at the tick feeding site and clear microbes in blood meal [54]. However, data in Fig. 4e show that putative AMPs in this study were suppressed (Figs. 4E1-5) in response to *B. burgdorferi* infection. This finding suggests that AMPs in this study might not be required for transmission of *B. burgdorferi*. This finding was surprising, as we would expect for the tick to secrete AMPs to prevent secondary bacterial infections at the feeding site. It is notable that in *B. burgdorferi* infected tick saliva, one AMP (EEC20127.1) was suppressed from 20-60-fold between 12-72 h tick-feeding periods when *B. burgdorferi* is likely transmitted [33, 34], but increased to comparable levels in saliva of replete fed ticks (Figs. 4E2). The observation that AMP levels were higher in replete fed (96-120 h fed) ticks, points to the possibility that secretion of AMPs was manipulated by *B. burgdorferi* transmission events, which ceased toward end of tick feeding when transmission was not active.

#### *Borrelia burgdorferi* infection responsive histamine binding proteins/ lipocalins

Lipocalins among other ligands can bind histamine and other molecules [102] and on this basis, lipocalins also referred to as tick histamine-binding proteins were postulated to play roles in tick evasion of host inflammation defense by binding histamine that is released at the tick-feeding site [103, 104]. It is notable that, of the eight lipocalins that were enhanced in saliva of *B. burgdorferi* infected ticks (Figs. 4F1-8), five were secreted within 12-24 h (Figs. 4F1-5) of tick attachment. This finding is interesting in that there is evidence that tick sensitivity to histamine declines as tick attachment stabilizes [105]. It is potentially possible that lipocalins that are secreted at high abundance during the first 12-24 h might sequester histamine and shield *B. burgdorferi* infected ticks from the deleterious effects of histamine during the attachment phase. We speculate that the two lipocalins that were secreted at high abundance in replete fed (96-120 h fed) infected ticks (Figs. 4F7 and 8) and those that were suppressed (Figs. 4F9-16) are not associated with transmission of *B. burgdorferi*. It is possible that cargo of lipocalins that were suppressed in infected ticks might be toxic against transmitted *B. burgdorferi.*

#### Immune-related proteins

We have identified 40 putative immune related proteins, seven of which were suppressed in *B. burgdorferi* infected ticks (SF5A and S1 Table). Some of the immune-related proteins that were enhanced in saliva of *B. burgdorferi* infected ticks include four 14-3-3-phospho-binding proteins that regulate multiple signaling pathways including cancer, apoptosis, cell cycle progression, autophagy, glucose metabolism, and cell motility [97]. Another notable group of proteins are the novel E3 ubiquitin ligases (NEL) that might suppress pattern-recognition triggered immunity as suggested by indirect evidence in some pathogenic bacteria evasion of immunity in plants [106]. If functional, the six of the seven tick NEL proteins that were enhanced in infected ticks will aide in colonization of the host by transmitted *B. burgdorferi* by suppressing pattern recognition triggered immunity.

Other findings include a peptidyl-prolyl cis-trans isomerase, which in microbes acts as virulence factor and a potential drug target [107] and annexin, which is being targeted in anti-parasitic therapeutics [108]. It is also interesting to note that there is indirect evidence that suggest that tick annexin might interfere in the host’s complement defense against tick feeding and transmitted *B. burgdorferi*. Mammalian annexins can bind C1q [109] and factor H [110], which respectively regulate the classical and alternative complement pathways. Since *B. burgdorferi* is sensitive to complement killing [111], is possible that tick annexin might serve as decoys to protect transmitted *B. burgdorferi* against complement killing. Other notable proteins enhanced with infection include immunoglobulin superfamily proteins that have been associated with immune regulation [112] and cyclophilin A was shown to enhance viral replication [113–115]. and it will be interesting to investigate the impact on transmitted *B. burgdorferi* at the tick-feeding site. Likewise, hemolectin is a haemocyte specific protein that is similar to von Willibrand factor and regulates hemolymph clotting and immunity in *Drosophila* [116, 117]. In mammals, the toll-like receptor 9 protein plays roles in both innate immunity such as pattern recognition [109], and adaptive immunity [118, 119]. Therefore, it is interesting to note that toll-like receptor 9-like proteins were secreted at high abundance by *B. burgdorferi* infected ticks: there is a possibility that these proteins could interfere with functions of pattern recognition receptors on immune cells and aide in host colonization by transmitted *B. burgdorferi.*

#### *Borrelia burgdorferi* infection responsive glycine rich proteins and extracellular matrix-like proteins

Supplemental figures, SF5B and SF5C summarizes the secretion dynamics of 10 glycine rich proteins (GRP) and 45 putative extracellular matrix-like (ECM) proteins, respectively. Of the 10 GRPs, three were enhanced, two were unmodified, and the remaining five were suppressed in response to *B. burgdorferi* infection. Our annotation of GRPs as ECMs was based on the fact that these proteins play roles in formation of the amorphous adhesive substance (tick cement) that glues the tick to host skin [120]. Since tick cement will be required by both uninfected and infected ticks, the two GRPs (XP_029830864.1 and EEC14463.1) that were unmodified with *B. burgdorferi* infection could be associated with tick cement formation, while those that were enhanced or suppressed perform functions outside tick cement formation.

Indirect evidence suggests that some of the ECM proteins in this study might directly interact with *B. burgdorferi.* Available data show that *B. burgdorferi* encodes a fibronectin binding protein [121] and that binding of plasma fibrinogen aide spirochetes to circulate in the blood stream [122]. On this basis it is logical to assume that, fibronectin proteins that were enhanced in *B. burgdorferi* infected ticks might aide transmitted *B. burgdorferi* to disseminate from its site of inoculation. It is also interesting to note that *B. burgdorferi* enhances expression of adhesion molecules by endothelial cells [123], and thus it is possible that the neural cell adhesion molecules that were enhanced in infected ticks might interact with transmitted *B. burgdorferi* at the tick-feeding site to initiate dissemination.

Other notable ECM-like proteins include putative chitinases, which when active will degrade chitin. Active chitinases are characterized by the functional domain FDG(L/F)DL**D**W**E**(Y/F)P, where the bolded “D” and “E” amino acids are important for the chitinase function [124, 125]. It is interesting to note that the functional domain is conserved in one of the five putative chitinases (XP_029843550.1) that were enhanced in infected ticks. This chitinase sequence show 53-65% amino acid identity to the *A. americanum* short and long chitinase isoforms (Accession # AIR95100.1 and AIR95099.1), which when disrupted by RNAi silencing loosened the tick cement cone resulting in bleeding around the tick attachment site [126]. There is indirect evidence in other parasites that suggest that chitinases might promote *B. burgdorferi* colonization of the host as indicated *Toxoplasma gondii* chitinase modulated functions of macrophages and promoted establishment of the parasite [127].

#### Housekeeping-like proteins

Of the 747 proteins in this study, 355 belong to 15 housekeeping and housekeeping-like protein functional categories (SF5D-R). Although functional studies are needed to establish if housekeeping-like proteins in this study are functional, secretion patterns of some of the housekeeping-like proteins suggest that *B. burgdorferi* infection influenced secretion patterns of tick saliva proteins to its benefit. We developed commentary on housekeeping-like proteins to highlight putative roles in *B. burgdorferi* transmission.

##### *Cytoskeletal* (CS)

A total of 98 CS proteins, 39 of which were suppressed with *B. burgdorferi* infection (SF5D). Majority of CS proteins that were enhanced in *B. burgdorferi* infected ticks were associated with actin function [128]. There is evidence that macrophage and dendritic cells phagocytosis of *B. burgdorferi* was actin dependent [129], while *B. burgdorferi* encoded actin was critical to its motility [130]. It will be interesting to investigate effect of tick actin on ability of macrophages and dendritic cells and on host colonization by transmitted *B. burgdorferi.*

##### *Detoxification*/*Antioxidants*

The ticks’ disruptive feeding style is expected to damage host tissues/cells that would accumulate reactive oxygen species (ROS) at the tick-feeding site. A total of 25 antioxidants were identified with majority (*n*=16) being enhanced and the rest suppressed (SF5E). Notable in our data is the fact that *B. burgdorferi,* which is susceptible to toxicity of reactive oxygen species (ROS) including hydrogen peroxide (H_2_O_2_) [131, 132], enhanced secretion of antioxidants that neutralize H_2_O_2_ and other ROS including catalase, thioredoxin, thioredoxin reductase, selenium, and glutathione S-transferase in *B*. *burgdorferi* infected tick saliva [133–135]. On the contrary copper/zinc superoxide dismutase, which produces H_2_O_2_ through catalyzing the two-step dismutation of superoxide radical (O_2_^-^) to O_2_ and H_2_O_2_ [136] was suppressed in saliva of *B*. *burgdorferi* infected ticks (SF5E). It is noteworthy that genes encoding for catalase and copper/zinc superoxide dismutase that were respectively enhanced and suppressed in *B. burgdorferi* infected saliva are absent in the *B. burgdorferi* genome [137]. There is evidence that *B. burgdorferi* resistance to oxidative stress is critical to their survival in the vertebrate host [131, 138–143]. In the vertebrate host, *B. burgdorferi* expresses different genes that mitigate the effects of the host’s oxidative antimicrobial defense. Whether or not *B. burgdorferi* antioxidant genes are expressed in real time as it is transmitted to the host by the tick is unknown.

##### Hemelipoproteins/vitellogenin, and ferritin

The secretion dynamics for hemelipoproteins and vitellogenin (*n*=25) and ferritin (*n*=2, highly identical with differences in the amino-terminus region) are shown in SF5. Except for the two ferritins and three hemelipoproteins that were present only in *B. burgdorferi* infected tick saliva, the remaining 22 proteins were enhanced, suppressed or unchanged in abundance (ST1). It is interesting to note that 21 of the 22 proteins were enhanced within the first 24 h of feeding in *B. burgdorferi* infected tick saliva. This finding suggests that hemelipoproteins might not perform functions that are unique to *B. burgdorferi* transmission or that they could regulate critical tick feeding functions that are essential to both uninfected and *B. burgdorferi* infected tick feeding. It is noteworthy that ferritin was only detected in infected ticks suggesting a role in *B. burgdorferi* transmission. While other bacteria need host-derived iron to replicate [144, 145], there is evidence that *B. burgdorferi* does not require iron to replicate [146]. There is also evidence that *B. burgdorferi* that was loaded with iron was susceptible to killing by H_2_O_2_ and human polymorphonuclear leukocytes [147]. Given that ferritin is an iron storage protein [148], it could be feasible that *B. burgdorferi* infection induces high secretion of ferritin to sequester iron at the feeding site to protect the transmitted *B. burgdorferi* from iron-triggered toxicity.

##### Metabolism associated proteins- nucleic acids

We identified 11 nucleic acid metabolism proteins (SF5G), majority of which were enhanced in saliva of *B. burgdorferi* infected ticks. One of the enhanced nucleic acid metabolism proteins is 5′-nucleotidase, which in mammals catalyzes hydrolysis of extracellular nucleotides [149, 150] is also secreted into snake venom [151] and is an inhibitor of platelet aggregation [152]. Of note, inhibition of platelet aggregation is critical for ticks to complete feeding in that when small wounds occur, similar to tick feeding lesions, the host responds by forming a platelet plug at the injury site [153, 154]. Therefore, secretion of 5′-nucleotidase will likely aide in feeding success and transmission of *B. burgdorferi.*

##### Metabolism associated proteins- nuclear and transcriptional regulation proteins

We also identified 11 putative histone proteins, five of which were enhanced in saliva of *B. burgdorferi* infected ticks (SF5H). Similarly, we also identified five transcription regulation proteins that were enhanced in infected ticks (SF5I). Beyond the role of histones in transcriptional regulation [155], extracellular histones are also associated with regulation of host defense including formation of neutrophil extracellular traps (NETs), involvement in inflammation response to injury, and thrombosis [156, 157]. Mice NETs had lethality against *Borrelia afzelii* but was not inhibited by addition of saliva of adult *I. ricinus* ticks that had completed feeding [158]. It will be interesting to investigate the impact of *B. burgdorferi* infection enhanced histones on NET defense against transmitted *B. burgdorferi*.

##### Metabolism associated proteins- amino acids

It is interesting to note that all amino acid associated metabolism-associated proteins were enhanced in saliva of *B. burgdorferi* infected ticks. Given that *B. burgdorferi* does not encode for *de novo* synthesis of amino acids [137], it is notable that the amino acid metabolism associated enzymes (SF5J) that were enhanced in saliva of *B. burgdorferi* infected ticks are involved in catabolism of amino acid residues. For instance, fumarylacetoacetase is involved in tyrosine catabolism [159], and dihydropteridine reductase is associated with hydroxylation of the aromatic amino acids (phenylalanine, tyrosine and tryptophan) for biosynthesis of neurotransmitters: dopamine, and serotonin [160, 161]. Aspartate aminotransferase catalyzes a forward reaction to degrade amino acids, and reverse reaction to synthesize aspartate [162]. Likewise, D-3-phosphoglycerate dehydrogenase enzyme is involved in serine amino acid residue synthesis [163]. It is possible that some these enzymes might aide transmitted *B. burgdorferi* to assimilate amino acids that are released at the tick-feeding site.

##### Metabolism associated proteins- carbohydrate and energy

**Fig. 5:**
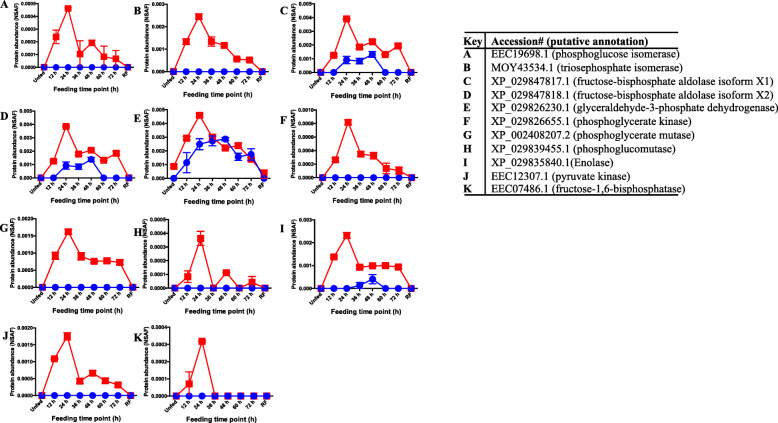
*Borrelia burgdorferi* infection of *Ixodes scapularis* nymph ticks induces secretion of glycolysis pathway enzymes. The normalized spectral abundance factor as the index for relative protein abundance of selected glycolysis pathway enzymes was graphed using PRISM8. The table insert lists accession numbers and putative annotations. The X-axis data points represent unfed stage, feeding time points every 12 h through 72 h, and replete-fed ticks. The Y-axis represents relative abundance as indicated by average NSAF values from the three LC-MS/MS runs

We respectively identified 16 and 41 proteins that were categorized as carbohydrate and energy metabolism proteins, respectively (SF5K and SF5L). It is notable that all carbohydrate metabolism associated proteins were enhanced in *B. burgdorferi* infected ticks (SF5K). Similarly, except for eight proteins, all energy metabolism associated proteins were enhanced with infection (SF5L). *B. burgdorferi* encodes the complete glycolytic pathway [137] that uses exogenous glucose and other carbohydrates to produce its ATP [164–166]. There is evidence that *B. burgdorferi* infection of the vertebrate host leads to enhanced glycolysis in immune cells [167]. Thus, it is interesting to note that *B. burgdorferi* infection of *I. scapularis* nymph ticks resulted in enhanced secretion of key glycolysis/gluconeogenesis pathway enzymes. To gain further insight into impact of *B. burgdorferi* infection on glycolysis/gluconeogenesis, the secretion dynamics as revealed by NSAF values (index for relative abundance) of glycolysis/ gluconeogenesis pathway enzymes are represented in Fig. 5. In the glycolysis pathway, glucose or other sugars are utilized to produce energy adenine triphosphate (ATP) and nicotinamide adenine dinucleotide hydride (NADH) the molecules that are needed for normal cellular function. On the flip side, gluconeogenesis is the reverse process of glycolysis that utilizes non-carbohydrate materials to make glucose [168]. The most notable in our dataset is that 9 of the 10 glycolysis pathway enzymes were enhanced in saliva of *B. burgdorferi* infected ticks (Fig. 5). These include glucose-6 phosphate-isomerase, fructose bisphosphate aldolase, and triosephosphate isomerase (Fig. 5) which represent the second, third, and last enzymes, respectively, in the first five-enzyme sequence that regulate the initial part of the glycolysis pathway referred to as energy consuming or preparatory phase. Interestingly, the complete set of the five enzymes that regulate the second part of glycolysis pathway referred to energy-producing phase: glyceraldehyde-3-phosphate dehydrogenase, phosphoglycerate kinase, phosphoglycerate mutase, enolase, and pyruvate kinase [169, 170] were secreted at high abundance by *B. burgdorferi* infected ticks (Fig. 5). Four enzymes: pyruvate carboxylase, phosphoenolpyruvate carboxykinase, fructose 1,6-bisphosphatase, and glucose 6-phosphatase are unique to gluconeogenesis pathway [171]. It is notable that fructose 1,6-bisphosphatase was secreted at high abundance by *B. burgdorferi* infected ticks (SF5L). Another notable observation in SF5K and SF5L is that all enzymes were highly secreted at the 24-48 h feeding time point preceding major events of *B. burgdorferi* transmission [33, 34].

Previous studies have shown that pyruvate can protect *B. burgdorferi* from H_2_O_2_ toxicity [172]. Thus, it is interesting to note that pyruvate kinase which catalyzes the last step of the glycolysis pathway yielding pyruvate and one molecule of ATP [173] was enhanced in *B. burgdorferi* infected saliva (Fig. 5). If functional in the extracellular space, both pyruvate and the synthesized ATP will benefit the transmitted *B. burgdorferi* from H_2_O_2_ toxicity*.* Another notable enzyme that was enhanced in saliva of infected ticks is alpha L-fucosidase: this enzyme cleaves off the fucose sugar molecule during the break down of complex sugars [174]. If functional, it is conceivable that alpha L-fucosidase might provide *B. burgdorferi* with access to simple carbohydrates that are yielded in degradation of complex sugars at the tick-feeding site. The *B. burgdorferi* encoded glycolysis pathway will then process these simple sugars to produce ATP needed by the transmitted *B. burgdorferi*.

##### Protein metabolism associated- heat shock proteins and others

We identified 60 protein metabolism associated proteins in six functional categories, majority of which were secreted at high abundance by infected suggesting roles in transmission of *B. burgdorferi*. The five functional categories include, protein modification associated (*n*=23, SF5M), protein export (*n*=5, SF5N), protein synthesis (*n*=9, SF5O), and proteasome machinery (*n*=9, SF5P), and transporter/receptor (*n*=9, SF5Q). The finding that housekeeping proteins (HSP) were secreted at high abundance by infected ticks is notable. There is evidence that *B. burgdorferi* secreted putative HSP are involved in regulating the spirochete’s interactions with its mammalian host immunity [175, 176]. Under normal physiology, HSPs are best known for their intracellular roles as stress response proteins and protecting cells from damage under stressful conditions [177, 178]. On the other hand, when secreted into the extracellular space, HSPs serve as alarm signals to the immune system and triggers injury repair responses including anti-inflammation [178]. Whether or not tick HSPs in this study are functional, remains to be determined. However, if functional, it is conceivable that tick HSPs might promote transmitted *B. burgdorferi* colonization of the host by serving as inhibitors of host inflammation response to tick feeding. It is interesting to note that similar to findings in this study, there is evidence that tick HSPs were enhanced or interacted with pathogens in ticks that were infected with tick-borne disease agents; *Anaplasma phagocytophilum* [29, 179, 180], and *Babesia bigemina* [181] and mammalian cell interaction with Crimean-Congo hemorrhagic fever virus [182, 183]. Interestingly, there is evidence that *I. scapularis* HSP70 (XP_002433656.1) in this study contributed to tick fibrinolysis activity [182, 184], which will promote *B. burgdorferi* dissemination.

##### Signal transduction

We identified 41 putative signaling/cell function associated housekeeping proteins that were responsive to *B. burgdorferi* infection (SF5R). Among proteins described here, calreticulin (CRT), which in mammals is a cytosolic protein that binds calcium, protein, and mRNA [184], is the most studied in tick physiology. Tick CRT is an immunogenic tick saliva protein that is currently used as the biomarker for tick bites [185–188]. There is also evidence that tick CRT is involved in regulating interactions between ticks and tick-borne disease agents in that RNAi silencing of tick CRT reduced the *Babesia bigemina* parasite load in *R. annulatus* [188]. We have previously shown that *A. americanum* tick CRT can bind C1q, which is part of the C1 complex, which activates the classical complement pathway [189]. Interestingly, tick CRT binding of C1q did not block complement activation, but it rather enhanced as addition of increased amounts of tick CRT resulted in increased deposition of the membrane attack complex. It is potentially possible that tick CRT serves as an activator of the classical complement pathway, and thus could serve as a decoy during *B. burgdorferi* transmission. It will be interesting to determine if the C1 complex preferentially deposits onto tick CRT than *B. burgdorferi* complement binding proteins*.* There is evidence that evasion of the classical complement pathway is required to regulate spirochetemia in mice [111]. It is also notable that while uninfected adult ticks secrete CRT during feeding [2, 24], CRT was secreted at detectable levels by *B. burgdorferi-*infected *I. scapularis* nymphs, but not the uninfected nymph. It will be interesting to investigate how tick CRT might protect *B. burgdorferi* from complement killing. Another interesting group of proteins are insulin growth factor binding proteins-related proteins (IGFBP-rP), which are part of the insulin and insulin-like growth factor signaling, which regulates multiple functions including immunity [190]. We found five IGFBP-rP sequences, three of which were suppressed, and two that were enhanced. We have recently shown that some of the tick IGFBP-rPs, which bind insulin [191], are involved in tick evasion of host inflammation defense [192]. Interestingly, RNAi silencing mediated disruption of *A. americanum* IGFBP-rP1 and IGFBP-rP6S and L significantly affected tick fitness and feeding success [193] demonstrating the significance of this protein in tick feeding physiology.

#### Tick-specific proteins of unknown function

Tick specific proteins of unknown function are so called because they did not match to proteins of other organisms in GenBank. We identified a total of 138 tick specific proteins, of which more than 70 were suppressed in response to *B. burgdorferi* infection (SF5S). Some of the most notable highlights in the data is the fact that nearly all tick salivary peptide group 1 and basic tails were suppressed in infected ticks. Very little is known about tick specific proteins in this study with exception of the AV422-like (XP_002406260.1), which is an *A. americanum* homolog that was highly up regulated in ticks that were stimulated to start feeding [194, 195] and might be considered as a marker for tick preparedness to start feeding. Thus, it is notable that the secretion level of XP_002406260.1 was not affected by *B. burgdorferi* infection suggesting that this protein regulates critical tick feeding functions. Interestingly, RNAi silencing of AV422 prevented *A. americanum* ticks from feeding to repletion [195].

##### Secretion dynamics of rabbit proteins in saliva of *Borrelia burgdorferi* infected ticks

Host derived proteins have been previously identified in tick saliva proteomes during tick feeding [24–26]. In this study, a total of 253 host-derived (rabbit) proteins were identified in saliva of *I. scapularis* uninfected and *B. burgdorferi* infected nymph saliva during feeding (ST1). The 253 host derived proteins were provisionally annotated into 21 functional categories. Of these, 12 (protease, protease inhibitors, keratin, metabolism of carbohydrates and energy, fibrinogen, signal transduction, globin/ RBC, transporter/ receptors, heme/ iron binding, lipocalin, and immune related proteins) were more abundant in saliva of *B. burgdorferi* infected ticks during feeding, four (metabolism of lipids, protein synthesis, transcription machinery, and nuclear regulation) were more abundant in uninfected tick saliva, and five (proteasome machinery, detoxification/ antioxidant, extracellular matrix, protein modification, and cytoskeletal) were variable in abundance or unchanged (SF6).

Presence of rabbit proteins in tick saliva might be dismissed as contamination. However, evidence in our data suggest that secretion of some the rabbit protein in tick saliva was influenced or induced by *B. burgdorferi* infection as indicated by the fact that majority of host proteins (52%) were secreted in *B. burgdorferi* infected ticks compared to 12% in uninfected ticks, and 36% in both treatment groups (ST1). It is interesting to note that the proteins in the 12 functional categories that were more abundant in *B. burgdorferi* infected tick saliva were present within the first 12 h of tick attachment, which could indicate role(s) of these proteins in conditioning the hosts’ feeding site for transmission of *B. burgdorferi*. It is noteworthy that except for the 12 h time point when protease inhibitors were secreted at low abundance by uninfected ticks, host derived proteases and protease inhibitors were present only in *B. burgdorferi* infected saliva (ST1) further demonstrating the impact of infection on secretion of rabbit proteins. Although present in both uninfected and *B. burgdorferi* infected tick saliva, the detoxification/antioxidant and extracellular matrix proteins were more abundant in uninfected ticks up to the first 24 h and then switched to abundance in saliva of infected ticks in the subsequent time points (SF6). As majority of *B. burgdorferi* transmission occurs around 48 h, tick saliva proteins that are reverted in abundance during feeding by *B. burgdorferi* infected ticks indicates that these proteins could play important roles in survival and facilitating transmission into the mammalian host. For instance, peroxiredoxin, which is absent in *B. burgdorferi* is an antioxidant of H_2_O_2_ and was identified only in *B. burgdorferi* infected tick saliva from 12-72 h of feeding (ST1) when transmission of the spirochete is expected [33, 45]. Since *B. burgdorferi* is sensitive to killing by H_2_O_2_ [131, 132], it is logical to assume that rabbit peroxiredoxin secreted in tick saliva could protect transmitted *B. burgdorferi.* It is also interesting to note that fibrinogen, which is the precursor to fibrin that forms a fibrin-based blood clot at the site of injury, was present only in *B. burgdorferi* infected tick saliva. Given that *B. burgdorferi* outer surface protein C (OspC), which binds fibrinogen [196] is up-regulated during spirochete transition from the midguts to the salivary glands [197], the finding that fibrinogen was secreted only in *B. burgdorferi* infected tick saliva could indicate its potential role(s) in transmission and dissemination through a hematogenous route.

#### *Borrelia burgdorferi* infected ticks provoke robust anti-tick immunity after a single infestation.

**Fig. 6:**
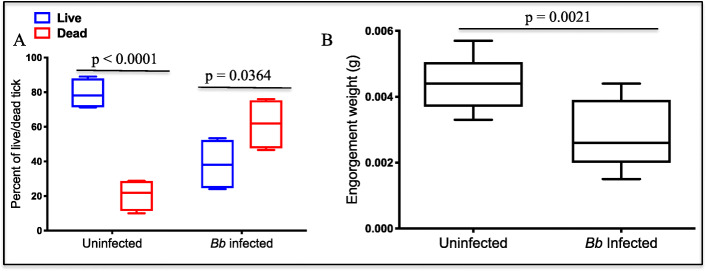
Single infestation of rabbits with *Borrelia burgdorferi* infected *Ixodes scapularis* nymph ticks elicits immunity against tick feeding. Three weeks after the primary infestation, New Zealand white rabbits were re-infested with uninfected and *Bb* infected *I. scapularis* nymphs (*n*=75). Tick feeding progress was monitored every 24 h; all attached (representing live ticks [blue box]) and detached (representing dead ticks [red box]) were counted. **a** Cumulative average of ticks that fed on four rabbits (*p*-value < 0.0001 when comparing live and dead from uninfected; *p*-value = 0.0364 when comparing live and dead from *B. burgdorferi* infected). **b** Mean engorgement weight (mg) of replete fed ticks (*p*-value = 0.0021 when comparing uninfected and *B. burgdorferi* infected). Unpaired t- test was used to determine statistical significance

Figure 6 summarizes a significant finding in this study, that a single infestation of rabbits by *B. burgdorferi* infected *I. scapularis* nymphs provoked protective anti-tick immunity that resulted in high mortality of *B. burgdorferi* infected ticks during the second infestation compared to uninfected ticks. Prompted by our finding that *B. burgdorferi* infected ticks secreted more tick saliva proteins at high abundance than uninfected ticks, we sought to repeatedly infest rabbits to elicit immunity against *B. burgdorferi* infection specific tick saliva proteins. To our surprise, although we did not observe any significant differences in tick feeding efficiency during the first infestation, a significantly high number of *B. burgdorferi* infected ticks died during the second infestation. We repeated this study with a cohort of 8 rabbits (4 each, uninfected and infected) and observed mortality of 46.5-76% of *B. burgdorferi* infected ticks compared to 15-28% for the uninfected ticks during the second infestation (Fig. 6a). Likewise, though not statistically significant, the engorgement mass as index for amount of blood that was ingested by *B. burgdorferi* infected ticks that fed to repletion was apparently smaller than uninfected ticks (Fig. 6b).

**Fig. 7:**
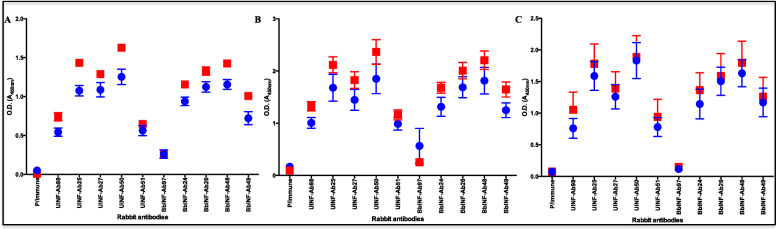
Tick saliva proteins eliciting immunity against *Ixodes scapularis* nymph feeding are apparently highly expressed in *Borrelia burgdorferi* infected ticks than uninfected. Total tick protein extracts (500 ng per well) of uninfected (blue) and *B. burgdorferi* infected (red) *I. scapularis* nymphs that fed on rabbits for 24, 48, and 72 h were subjected to standard ELISA using purified IgG (100 μg/mL) of rabbits that were exposed once (Ab97 and 98) or twice (Ab 24, 25, 26, 27, 48, 49, 50 and 51) to uninfected and infected ticks. Y-axis *= A*_450nm_, X-axis shows different rabbit antibodies. P/immune = pre-immune, UINF-Ab98, 25, 27, 50, and 51 = rabbit immune sera to tick saliva proteins of uninfected nymphs. BbINF-Ab97, 24, 26, 48, and 49 = rabbit immune sera to tick saliva proteins of *B. burgdorferi* infected nymphs. A, B, and C = ticks that fed for 24, 48, and 72 h respectively

Given the finding in Fig. 6, we were curious about antibody response to tick saliva proteins of uninfected and *B. burgdorferi* infected ticks (Fig. 7). We subjected protein extracts of whole uninfected and *B. burgdorferi* infected *I. scapularis* nymphs that fed on rabbits for 24 h (Fig. 7a), 48 h (Fig. 7b), and 72 h (Fig. 7c) to ELISA analysis using purified IgG of rabbits that were infested by uninfected (blue symbols) and *B. burgdorferi* infected (red symbols) ticks. Although not statistically significant as revealed by unpaired t-test, ELISA analysis shows that tick saliva proteins that elicited immunity against nymph tick feeding were apparently expressed at high level in *B. burgdorferi* infected ticks than uninfected (Fig. 7a-c). We would like to note that rabbit (antibody) 98 and 97 were infested once with uninfected and *B. burgdorferi* infected nymphs, respectively.

## Discussion

This study has described tick saliva proteomes of uninfected and *B. burgdorferi* infected *I. scapularis* nymphs*.* Data here clearly demonstrate that *B. burgdorferi* infection influences *I. scapularis* nymph ticks to secrete a high number of proteins than uninfected ticks. Our strategy to identify tick saliva proteins secreted by unfed ticks, and of fed ticks every 12 h during feeding allowed us to relate the secretion dynamics to transmission biology of *B. burgdorferi.* These data will allow us to prioritize tick saliva proteins that are secreted prior to, or during major transmission events of *B. burgdorferi* [33, 34] in our quest to identify target antigens for vaccines to prevent LD.

Since at the unfed stage, *B. burgdorferi* is predominantly resident in tick midguts and transmission events ramps up after 48 h of tick attachment [33, 34], it is logical to expect that *B. burgdorferi* might have no impact on protein composition in saliva of unfed ticks. Thus, it was interesting to note that we identified 75 more proteins in saliva of unfed *B. burgdorferi* infected than uninfected (Fig. 1). Given that *B. burgdorferi* infection of ticks in this study was done by capillary feeding, it is possible that an unusually high number of spirochetes than naturally infected ticks was delivered to midguts. If this is the case, it is plausible that some of the spirochetes might have migrated to the salivary glands impacting secretion dynamics of tick saliva proteins. It is also possible that the high number proteins secreted by unfed *B. burgdorferi* infected ticks might reflect the yet unknown impact of *B. burgdorferi* to condition the vertebrate host to physiology. We speculate that proteins identified in saliva of unfed *B. burgdorferi* infected ticks might be associated with conditioning of the host to promote *B. burgdorferi* colonization of the host. It is also notable that secretion of majority of tick saliva proteins by *B. burgdorferi* infected ticks ceased at the 72 h feeding time point when transmission of *B. burgdorferi* is expected to be completed or near completion [33, 34]. In contrast, replete fed (96-120 h fed) uninfected ticks secreted 124 more proteins than *B. burgdorferi* infected ticks (Fig. 1). Also, notable, functional categories that were enhanced in response to *B. burgdorferi* infection were secreted at the highest abundance during the first 24-48 h of tick feeding (Figs 2, 4, and 5; SF5), which precedes major *B. burgdorferi* transmission events that occur after the tick has attached for more than 48 h [33, 34].

Although not yet demonstrated, evidence that flaviviruses might be sequestered into exosomes during transmission [198] indicates that tick exosomes are secreted into the host during tick feeding. Multiple tick saliva proteins that were enhanced in saliva of *B. burgdorferi* infected ticks have been reported in mammalian and parasite exosomes [198–203]. For instance, serpins were reported in mammalian exosomes [199, 200], while cysteine proteases, fatty acid-binding protein, housekeeping-like proteins such as HSP and actin, glycolysis/gluconeogenesis enzymes such as enolase, antioxidants such as thioredoxin, and glutathione S-transferase were reported in helminth parasite exosomes [202, 203]. This might suggest the possibility that some of the tick saliva proteins that were secreted at high abundance in saliva of *B. burgdorferi* infected ticks might be associated with tick saliva exosomes.

It is important to acknowledge the limitation of this study. The most significant is that *B. burgdorferi* infected ticks used in this study were artificially infected. There is potential that ticks used in this study might have contained more spirochetes than what is normally observed in naturally infected ticks, and this might have affected secretion of some of the tick saliva proteins in this study. In future studies, it will be imperative to validate our findings in naturally *B. burgdorferi* infected ticks by feeding larvae on *B. burgdorferi* infected hosts and then validating protein secretion in molted infected nymphs. It is also important to note that we did not include a killed *B. burgdorferi* control. This treatment might have revealed tick saliva proteins in this study that could be present due to stress response. However, we are encouraged by findings in this study that the secretion dynamics of some of the proteins were not affected by *B. burgdorferi* infection of ticks. Of note we found that proteins for which secretion dynamics was unaffected by *B. burgdorferi* infection included homologs to AV422 and tick IGFBP-rP1; which we previously found among tick proteins that were enhanced in ticks that were stimulated to start feeding: on cattle [194], rabbits, dogs, and humans [22]. Therefore, the observations that AV422 and IGFBP-rP1 were unaffected by *B. burgdorferi* infection demonstrated the importance of these proteins in regulating tick feeding functions of both uninfected and *B. burgdorferi* infected ticks.

Another limitation of this study is the fact that ticks feeding on different hosts might utilize different tick saliva proteins. We have previously shown that both *Amblyomma americanum* and *I. scapularis* have different tick saliva protein profiles when stimulated to start feeding on different hosts [22]. Although there is a core set of proteins that are secreted into tick saliva regardless of the host [22], it is potentially possible that some of the tick saliva proteins identified in this study might not be secreted if ticks are fed on different host such as rodents or humans.

It is also important to note that several *Borrelia* spp and strains have been described [199], and it is potentially possible that tick saliva protein profiles will change depending on the species and/or strain of the infecting spirochete. Here, we used the wild type *B. burgdorferi* strain 31 MSK5 isolate which contains all relevant plasmids that promote infectivity [200]. Therefore, there is a high likelihood that majority of tick saliva proteins in this study will be associated with transmission of *B. burgdorferi* to humans.

## Conclusions

Given that *I. scapularis* nymphs transmits *B. burgdorferi* to majority of humans, data here provides the foundation upon which a tick-antigen based vaccine to prevent LD can be developed. Comparative analysis between uninfected and *B. burgdorferi* infected nymph tick saliva proteomes allowed us to identify three categories of tick saliva proteins: (i) enhanced, (ii) unmodified, or (iii) suppressed in response to *B. burgdorferi* infection. We speculate that tick saliva proteins that were enhanced in saliva of *B. burgdorferi* infected ticks play significant roles in transmission of *B. burgdorferi* and represent important targets for developing tick antigen-based vaccines to LD*.* Equally important, tick saliva proteins that were unmodified with *B. burgdorferi* infection likely regulate key tick feeding functions, which if blocked will disrupt feeding physiology and prevent transmission of *B. burgdorferi*. Lastly, we think that tick saliva proteins that were suppressed in response to *B. burgdorferi* infection are likely not good targets for tick vaccine development. However, some of these proteins might have lethality against transmitted *B. burgdorferi* and thus might be a source for design of novel anti-*B. burgdorferi* therapeutics.

## Methods

### Ethics statement

All experiments were done according to the animal use protocol approved by Texas A&M University Institutional Animal Care and Use Committee (IACUC) (AUP 2017-0068 and 2018-0001) that meets all federal requirements, as defined in the Animal Welfare Act (AWA), the Public Health Service Policy (PHS), and the Humane Care and Use of Laboratory Animals. This study used 12 rabbits, all of which were processed for terminal bleed collections under general anesthesia as approved by the AVMA (American Veterinary Medical Association) and adopted by Texas A&M University IACUC.

### Infecting *Ixodes scapularis* nymphs with *Borrelia burgdorferi*

*Ixodes scapularis* nymph ticks used in this study were purchased from the tick laboratory at Oklahoma State University (Stillwater, OK, USA). *Ixodes scapularis* nymphs were infected with *B. burgdorferi* (>1x10^7^ cells/ml) cultured in BSK-H (Sigma-Aldrich, St Louis, MO) complete media supplemented with 6% rabbit serum by capillary feeding methods using as described with modifications [35]. The wild type *B. burgdorferi* strain 31 MSK5 isolate that was used in this study contains all relevant plasmids for infectivity [204] and was initially isolated from tick midguts [205]. The *B. burgdorferi* (strain B31 clone MSK5) that was used in this study was kindly gifted by Dr. Jon T. Skare (TAMU Health Science Center) and was routinely propagated in BSK-H complete media. To prepare *B. burgdorferi* for feeding to nymphs, 10 mL of BSK-H culture of log-phase spirochetes were pelleted at 300g for 10 min at room temperature (RT) and then re-suspended in 1 mL of fresh BSK-H media to yield >1x10^7^ cells/ml. To prepare the feeding unit, a sterile 10 μL pipette filled with 5 μL of the *B. burgdorferi* concentrated culture was taped onto a glass slide. Under a dissecting scope, the tick's mouthpart was inserted into the pipette tip opening. To prevent ticks from dislodging, the feeding unit was inserted into another modified pipette tip as shown in SF1A. Based on an empirically determined timeline, nymphs attached to the feeding unit were incubated in a humidified chamber in an incubator at 32°C with 1% CO_2_ for 1-2 h to allow for feeding to complete. After feeding, ticks were kept at 22-24°C with ~90% humidity for one week to allow for *B. burgdorferi* to adapt to the tick environment before proceeding to tick feeding on rabbits and collecting tick saliva (detailed below).

Routinely, for every batch of *B. burgdorferi*-infection, 10% of ticks were processed to confirm *B. burgdorferi* infection using *fla*B gene primers [206]. Ticks were disinfected by rinsing with 1% bleach, 70% ethanol, and water (repeated 3X for each solution) and then individually processed for genomic DNA extraction using the DNAeasy Blood and Tissue Kit (Qiagen, Hilden, Germany). The genomic DNA was used in qualitative PCR using *fla*B gene (GenBank: KR782218.1) forward (5’-CACATATTCAGATGCAGACAGAGGTTCTA-3’) and reverse (5’- AATTGCATACTCAGTACTATTCTTTATAGAT-3’) primers. We did not proceed with saliva collection (described below) if infection was below 80%. Subsequently, we collected sera of infested rabbits to validate transmission of *B. burgdorferi* by ELISA and western blotting analyses of laboratory cultured tick extracts.

### Collecting *Ixodes scapularis* nymph tick saliva

Uninfected or *B. burgdorferi*-infected *I. scapularis* nymph ticks were fed on 10-12 weeks old New Zealand white female rabbits (*n*=2 per treatment group for total of 4, Harlan Sprague Dawley Inc., Indianapolis, IN, USA). Tick feeding was done as published [54] and approved in animal use protocols 2017-0068 and 2018-0001. Ticks were restricted to feed on top of the rabbit ear using the two-inch cotton stockinet tick containment cells that were glued onto rabbit ears using the Kamar adhesive (Kamar Products Inc., Zionsville, IN, USA). Given the small size of nymphs, the tick containment cell was lined with Pantyhose material to prevent ticks from escaping. Saliva was collected from uninfected and *B. burgdorferi*-infected *I. scapularis* unfed nymphs (*n*=50) and partially fed nymphs (*n*=30 per time point) for 12, 24, 36, 48, 60, and 72 h (manually detached), and replete fed nymphs (*n*=15) ticks. To collect tick saliva, we first aspirated 5 μL of 2% pilocarpine-PBS solution into a 10 μL pipette tip, which were subsequently taped onto a glass slide. Under a dissecting scope, tick mouthparts were inserted into pilocarpine-PBS solution in the pipette tip as illustrated in SF1. To secure the nymph during saliva collection, a protective cap was prepared by modifying a 10 μL pipette tip. The tick saliva collection unit was then placed in humidified chamber at RT for 4 h (empirically determined during preliminary studies) with visual inspection every 30 min to check for disengaged ticks.

### LC-MS/MS analysis of tick saliva proteins

*I. scapularis* nymph tick saliva proteins were identified using LC-MS/MS analysis as previously described [22, 24, 26]. Approximately 2 μg of total tick saliva proteins (in triplicate) per feeding time point was processed using LC-MS/MS. Briefly, proteins were digested overnight at 37C using trypsin (Promega, Madison, WI, USA) at 1:20 (enzyme:protein) ratio in 2 M urea/0.1M Tris pH 8.5, 1 mM CaCl_2_ reaction buffer. Subsequently, the digested peptides were processed for LC-MS/MS analysis [22, 24, 26] using the nanoflow liquid chromatography mass spectrometry using a Q Exactive mass spectrometer (Thermo Fisher Scientific).

### Identification of tick saliva proteins

Tandem mass spectra that was extracted using the RawExtract 1.9.9.2 [207] were searched against the protein database using ProLuCID in the Integrated Proteomics Pipeline [208]. The protein database was assembled from protein sequences of tick and spirochete (GenBank), rabbit (Uniprot), and decoys (reverse sequences of all entries). Redundancies in the database were eliminated at 98% amino acid identity using the CD-hit program [209]. With false discovery rate (FDR) set to 1% hit to decoys, proteins with at least two six residue peptides hits in 2 of the 3 runs were accepted. Results were post processed to only accept peptide/spectrum matches (PSM) with <10ppm precursor mass error. Finally, the Identification Compare (IDcompare) program on IP2 Pipeline [208] was used to combine protein matches from each time points into one file.

For annotation of tick proteins were searched against multiple databases including GenBank, Uniprot [210], MEROPS database [42], conserved domains at NCBI, and SWISS-MODEL homology modeling at ExPASY [211]. The outputs from the blast searches were loaded into the classifier program in Dr. Jose M. Ribeiro's visual basic program [212] for functionally categorization.

### Graphic visualization of secretion dynamics of *Ixodes scapularis* tick saliva proteins

The normalized spectral abundance factors (NSAF), which is validated to estimate relative abundance in label-free quantification in LC-MS/MS analysis of protein [38–40] was used to determine relative abundance of proteins at every time point. To gain insights into secretion dynamics of functional categories, NSAF sum totals expressed as percentage for each functional category were normalized using Z-score statistics as described [22, 24, 26]. Subsequently, heatmaps of normalized percent NSAF were generated using the heatmap2 function in gplots library in R [41]. The secretion dynamics (low to high abundance) were used to assemble clusters on heatmaps. To gauge insight into secretion dynamics of the individual proteins, average NSAF values (at least two of the three runs) of individual proteins were graphed using PRISM 8 (GraphPad Software, San Diego, CA).

### Effect of repeated infestation on tick feeding success

This experiment was prompted by findings in this study that *B. burgdorferi* infected nymphs were secreting more proteins at high abundance than uninfected ticks. Our goal was to provoke high antibody titers to tick saliva proteins that were secreted by *B. burgdorferi* infected ticks. To our surprise, we observed significantly high mortality of *B. burgdorferi* infected ticks during the second infestation than the uninfected. To validate these observations, eight rabbits divided in two cohorts of four rabbits each were repeatedly infested twice three weeks apart with uninfected and *B. burgdorferi* infected ticks (*n*=70 per rabbit). Tick attachment was confirmed after 24 h and all unattached ticks were removed. Thereafter, live (attached and feeding) and dead ticks (detached) were counted every 24 h. We calculated the mortality rate as a percentage of dead ticks over ticks that were confirmed attached at 24 h after ticks were placed on rabbits. Ticks that completed feeding were weighed individually to determine the engorgement mass as index for amount of blood that was ingested. Rabbit blood collections were obtained before tick infestation (pre-immune) and after completion of uninfected and *B. burgdorferi* infected tick feeding every week for 4 weeks. Sera was used to confirm if rabbits seroconverted by western blot and ELISA analyses.

### ELISA to quantify immunogenic tick saliva protein expression

Prompted by findings of high mortality of *B. burgdorferi* infected ticks during second infestation, we sought to investigate antibody titers to tick saliva proteins in rabbits that were infested by uninfected and *B. burgdorferi* infected ticks using standard ELISA. Total proteins of whole uninfected and *B. burgdorferi* infected ticks (pools of 10 ticks per time point): unfed and fed on rabbits for 24, 48, and 72 h were minced using soft tissue scissors and processed for total protein extraction by sonication on ice in denaturing buffer (8M Urea, 100 mM Tris, 150 mM NaCl, 1% SDS, 5mM DTT, pH 7.4). After centrifugation to pellet non-dissolved tissues, the supernatant was filter sterilized (0.2 μm) and used for total protein quantification using the BCA method (Thermo Scientific). Prior to BCA quantification, the samples were diluted 50-fold to reduce the potential background from Urea, SDS, and DTT.

A 96-well plate was coated with 500 ng of total whole tick protein per well and subjected to standard ELISA analysis using purified IgG (100 μg/ml) of rabbits that were infested with uninfected or *B. burgdorferi* infected *I. scapularis* nymphs, followed by goat anti-rabbit IgG-HRP secondary antibody (1:5000). Using purified IgG allowed us to utilize equivalent amount of antibody in our ELISA and get insight into relative antibody titer levels. Purification of IgG was done using the protein G column according to instructions by the manufacturer (GE Healthcare). The positive signal was detected using the 1-Step Ultra TMB-ELISA substrate (Thermo Scientific). With background removed, OD in triplicate were plotted in PRISM 8.

### Validate tick transmission of *Borrelia burgdorferi* to rabbits

To validate if ticks artificially infected by capillary feeding were transmitting to rabbits, lab cultured *B. burgdorferi* was subjected to standard ELISA and western blotting analysis. The second passage of *B. burgdorferi* culture was harvested by centrifugation at 300g for 10 min at RT. Following washing of the pellet in phosphate buffered saline (PBS) to remove excess culture media components, total *B. burgdorferi* proteins was extracted by sonication in PBS on ice. The supernatant was filter sterilized (0.2 μm) and quantified using the BCA method (Thermo Scientific). Subsequently, 1 and 3 μg of total *B. burgdorferi* protein extract was respectively subjected to ELISA and western blotting analyses using purified immune IgG. For western blotting analysis, *B. burgdorferi* protein extract was resolved on a 10-20% Tris-Glycine SDS-PAGE and transferred onto a PVDF membrane. After blocking in 5% skim milk in PBS w/ Tween-20 (0.05%), the plates or membranes were exposed to the purified IgG (10 μg/ml) from pre-immune and immune sera of rabbits that were infested by uninfected ticks (*n*=5) and *B. burgdorferi* infected (*n*=5). To visualize the positive signals, a goat anti-rabbit IgG-HRP secondary antibody (1:5000) and 1-Step Ultra TMB-ELISA substrate (Thermo Scientific) or SuperSignal West Femto Maximum Sensitivity Substrate (Thermo Scientific) were used.

## Supplementary Information

**Additional file 1: SF1.** Non-invasive method of collecting saliva from *Ixodes scapularis* nymphs. Tick saliva collections were performed using a 10μl pipette tip set up. A modified 10μl pipette tip was used to affix the tick mouthpart in the solution and restrict the tick from escaping. Saliva collections from ticks were not included if leakage of fluid was detected around the protective cap. **SF2.** Antibody response *to Borrelia burgdorferi* antigens by ELISA and western blotting analyses. Total protein extracts from *B. burgdorferi* (1 or 3 μg) were coated per well for ELISA (A) or resolved by SDS-PAGE for western blotting (B) analyses using purified IgG (10μg/ ml) from pre-immune (PI), rabbit antibody (Ab) numbers 98, 25, 27, 50 and 51 from rabbits that were infested with uninfected nymphs and Ab numbers 97, 24, 26, 48, and 49 from rabbits that were infested with *B. burgdorferi* infected nymphs. For ELISA, the y-axis represents the A_450_ and x-axis represent the rabbit number. **SF3.** Profile of uninfected and *Borrelia burgdorferi* infected *Ixodes scapularis* nymph tick saliva proteins during feeding. Uninfected and *B. burgdorferi* infected *I. scapularis* nymph ticks that were unfed, partially fed for 12, 24, 36, 48, 60, and 72h, and replete-fed, were stimulated to salivate by injecting 2% pilocarpine into hemolymph. Saliva was electrophoresed on a 10-20% acrylamide gel and silver stained. Please note the molecular weight ladder from 10-250kDa. **SF4.** Secretion dynamics of all 747 proteins identified in uninfected and *Borrelia burgdorferi* infected *Ixodes scapularis* nymph tick saliva. Normalized spectral abundance factors (NSAF) values of all *I. scapularis* nymph tick saliva proteins identified in this study were normalized using the z-score statistics and then used to generate heat maps using heatmap2 function in gplots library using R as described in materials and methods. The red color represents high abundance to blue color indicating low abundance. **SF5**. Secretion dynamics of protein categories identified in uninfected and *Borrelia burgdorferi* infected *Ixodes scapularis* nymph tick saliva. Normalized spectral abundance factors (NSAF) values of *I. scapularis* nymph tick saliva proteins grouped in categories were normalized using the z-score statistics and then used to generate heat maps using heatmap2 function in gplots library using R as described in materials and methods. The red color represents high abundance to blue color indicating low abundance. A- immune related, B- glycine rich, C- extracellular matrix, D- cytoskeletal, E- detoxification/ antioxidant, F- heme/iron binding, G- nucleic acid metabolism, H- nuclear regulation, I- transcription machinery, J- amino acid metabolism, K- carbohydrate metabolism, L- energy metabolism, M- protein modification, N- protein export, O- protein synthesis, P- proteasome machinery, Q- transporters/receptors, R- signal transduction, and S- tick-specific saliva proteins of unknown function. **SF6.** *Borrelia burgdorferi* (*Bb*) infection modifies protein content on composition of rabbit (host) proteins in *Ixodes scapularis* nymph saliva. Cumulative normalized spectral abundance factor (NSAF) value, the index for relative protein abundance for all rabbit (host) proteins in saliva of uninfected and *Bb* infected nymph ticks was normalized using the z-score statistics and then used to generate heat maps using heatmap2 function in gplots library using R as described in materials and methods. The red to blue transition denotes high to low abundance levels shown in the Z-score range key. The reader is advised that the raw NSAF values that were used to generate the heatmap are provided in S1 Table.

**Additional file 2.**

**Additional file 3.**

## Data Availability

The mass spectrometry proteomics data have been deposited to the ProteomXchange Consortium via the PRIDE partner repository with the dataset identifier PXD023940 and 10.6019/PXD023940. Please note that ST1 includes GenBank accession numbers for tick proteins and Uniprot accession numbers for rabbit proteins.

## Abbreviations

LD: Lyme disease
Bb: *Borrelia burgdorferi*
LC-MS/MS: Liquid chromatography-tandem mass spectrometry
BSK-H: Barbour-Stoenner-Kelly-H
RT: Room temperature
NSAF: Normalized spectral abundance factors
SDS: Sodium dodecyl sulfate
DTT: Dithiothreitol
IgG: Immunoglobulin G
Serpin: Serine protease inhibitor
TIL: Trypsin inhibitor-like
MP: Metalloprotease
AMP: Antimicrobial peptides
TSP: Tick-specific proteins of unknown function
RNAi: RNA interference
PAR: Protease-activated receptors
NPC: Niemann-Pick type C
ML: MD-2-related lipid-recognition
GRP: Glycine rich proteins
ECM: Extracellular matrix
CS: Cytoskeletal
ROS: Reactive oxygen species
H_2_O_2_: Hydrogen peroxide
NET: Neutrophil extracellular traps
ATP: Adenine triphosphate
NADH: Nicotinamide adenine dinucleotide hydride
HSP: Heat shock protein
CRT: Calreticulin
IGFBP-rP: Insulin growth factor binding proteins-related proteins
OspC: Outer surface protein C
ELISA: Enzyme-linked immunosorbent assay
