# Supplementary figures and images for: Borrelia burgdorferi infection modifies protein content in saliva of Ixodes scapularis nymphs
Source: BMC Genomics. 2021 Mar 4;22:152. doi: 10.1186/s12864-021-07429-0 (PMC7930271; doi:10.1186/s12864-021-07429-0)

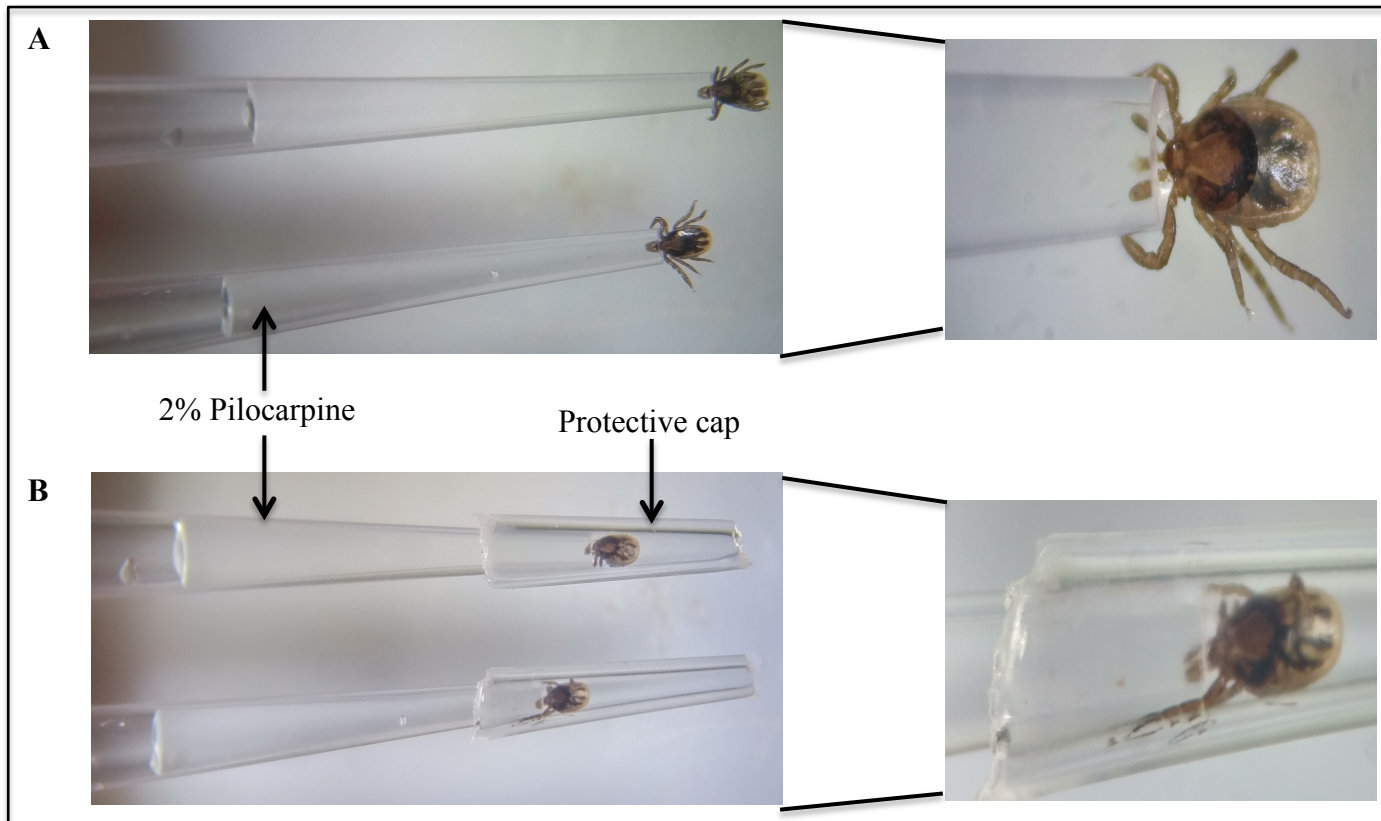

**Supplemental figure 1**

Supplement: Supplementary file 1 — Additional file 1: SF1. Non-invasive method of collecting saliva from Ixodes scapularis nymphs. Tick saliva collections were performed using a 10μl pipette tip set up. A modified 10μl pipette tip was used to affix the tick mouthpart in the solution and restrict the tick from escaping. Saliva collections from ticks were not included if leakage of fluid was detected around the protective cap. SF2. Antibody response to Borrelia burgdorferi antigens by ELISA and western blotting analyses. Total protein extracts from B. burgdorferi (1 or 3 μg) were coated per well for ELISA (A) or resolved by SDS-PAGE for western blotting (B) analyses using purified IgG (10μg/ ml) from pre-immune (PI), rabbit antibody (Ab) numbers 98, 25, 27, 50 and 51 from rabbits that were infested with uninfected nymphs and Ab numbers 97, 24, 26, 48, and 49 from rabbits that were infested with B. burgdorferi infected nymphs. For ELISA, the y-axis represents the A450 and x-axis represent the rabbit number. SF3. Profile of uninfected and Borrelia burgdorferi infected Ixodes scapularis nymph tick saliva proteins during feeding. Uninfected and B. burgdorferi infected I. scapularis nymph ticks that were unfed, partially fed for 12, 24, 36, 48, 60, and 72h, and replete-fed, were stimulated to salivate by injecting 2% pilocarpine into hemolymph. Saliva was electrophoresed on a 10-20% acrylamide gel and silver stained. Please note the molecular weight ladder from 10-250kDa. SF4. Secretion dynamics of all 747 proteins identified in uninfected and Borrelia burgdorferi infected Ixodes scapularis nymph tick saliva. Normalized spectral abundance factors (NSAF) values of all I. scapularis nymph tick saliva proteins identified in this study were normalized using the z-score statistics and then used to generate heat maps using heatmap2 function in gplots library using R as described in materials and methods. The red color represents high abundance to blue color indicating low abundance. SF5. Secretion dynamics of pro [file 12864_2021_7429_MOESM1_ESM.zip › SF1_ESM.pdf]

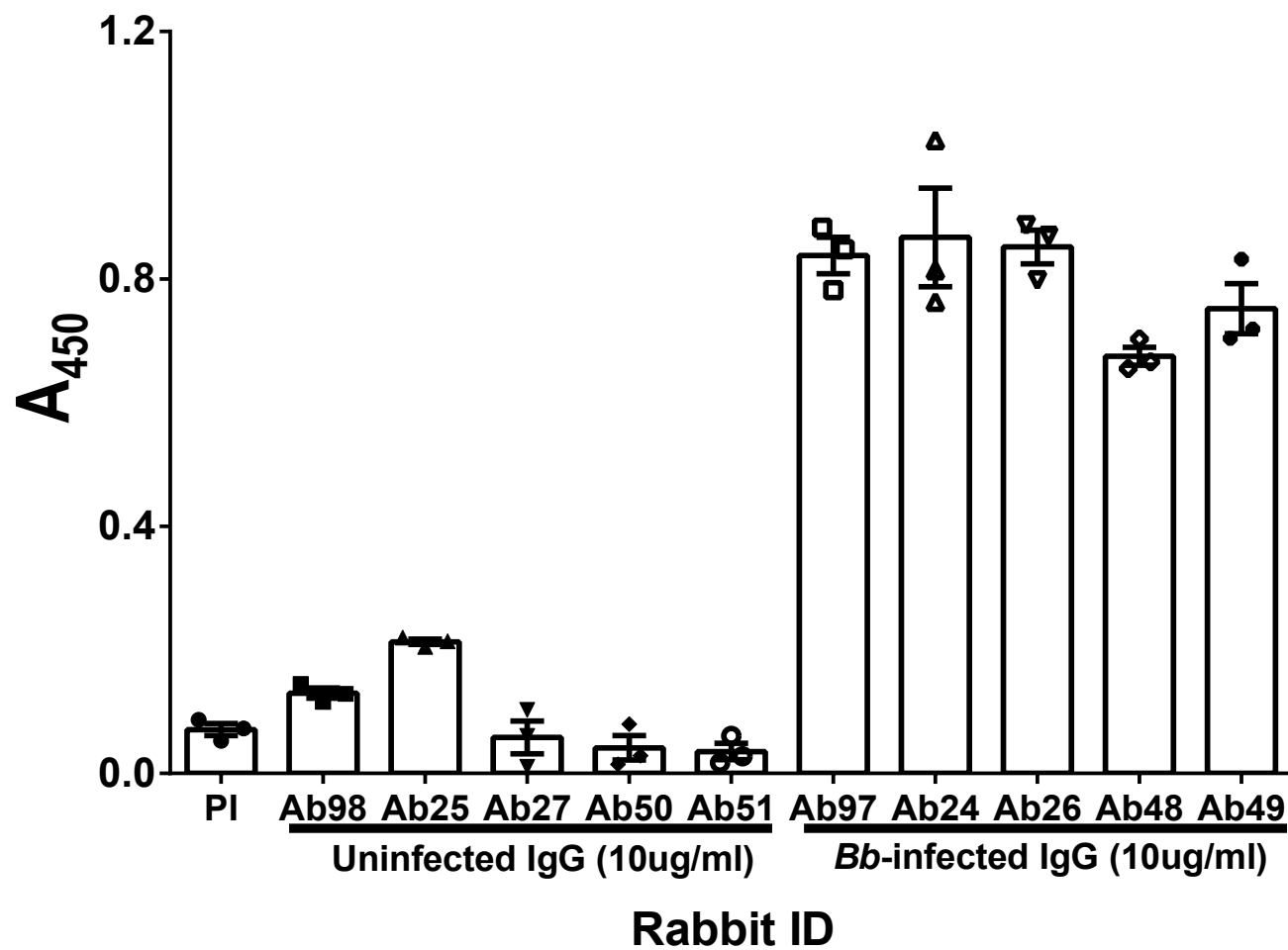

Supplement: Supplementary file 1 — Additional file 1: SF1. Non-invasive method of collecting saliva from Ixodes scapularis nymphs. Tick saliva collections were performed using a 10μl pipette tip set up. A modified 10μl pipette tip was used to affix the tick mouthpart in the solution and restrict the tick from escaping. Saliva collections from ticks were not included if leakage of fluid was detected around the protective cap. SF2. Antibody response to Borrelia burgdorferi antigens by ELISA and western blotting analyses. Total protein extracts from B. burgdorferi (1 or 3 μg) were coated per well for ELISA (A) or resolved by SDS-PAGE for western blotting (B) analyses using purified IgG (10μg/ ml) from pre-immune (PI), rabbit antibody (Ab) numbers 98, 25, 27, 50 and 51 from rabbits that were infested with uninfected nymphs and Ab numbers 97, 24, 26, 48, and 49 from rabbits that were infested with B. burgdorferi infected nymphs. For ELISA, the y-axis represents the A450 and x-axis represent the rabbit number. SF3. Profile of uninfected and Borrelia burgdorferi infected Ixodes scapularis nymph tick saliva proteins during feeding. Uninfected and B. burgdorferi infected I. scapularis nymph ticks that were unfed, partially fed for 12, 24, 36, 48, 60, and 72h, and replete-fed, were stimulated to salivate by injecting 2% pilocarpine into hemolymph. Saliva was electrophoresed on a 10-20% acrylamide gel and silver stained. Please note the molecular weight ladder from 10-250kDa. SF4. Secretion dynamics of all 747 proteins identified in uninfected and Borrelia burgdorferi infected Ixodes scapularis nymph tick saliva. Normalized spectral abundance factors (NSAF) values of all I. scapularis nymph tick saliva proteins identified in this study were normalized using the z-score statistics and then used to generate heat maps using heatmap2 function in gplots library using R as described in materials and methods. The red color represents high abundance to blue color indicating low abundance. SF5. Secretion dynamics of pro [file 12864_2021_7429_MOESM1_ESM.zip › SF2A_ESM.pdf]

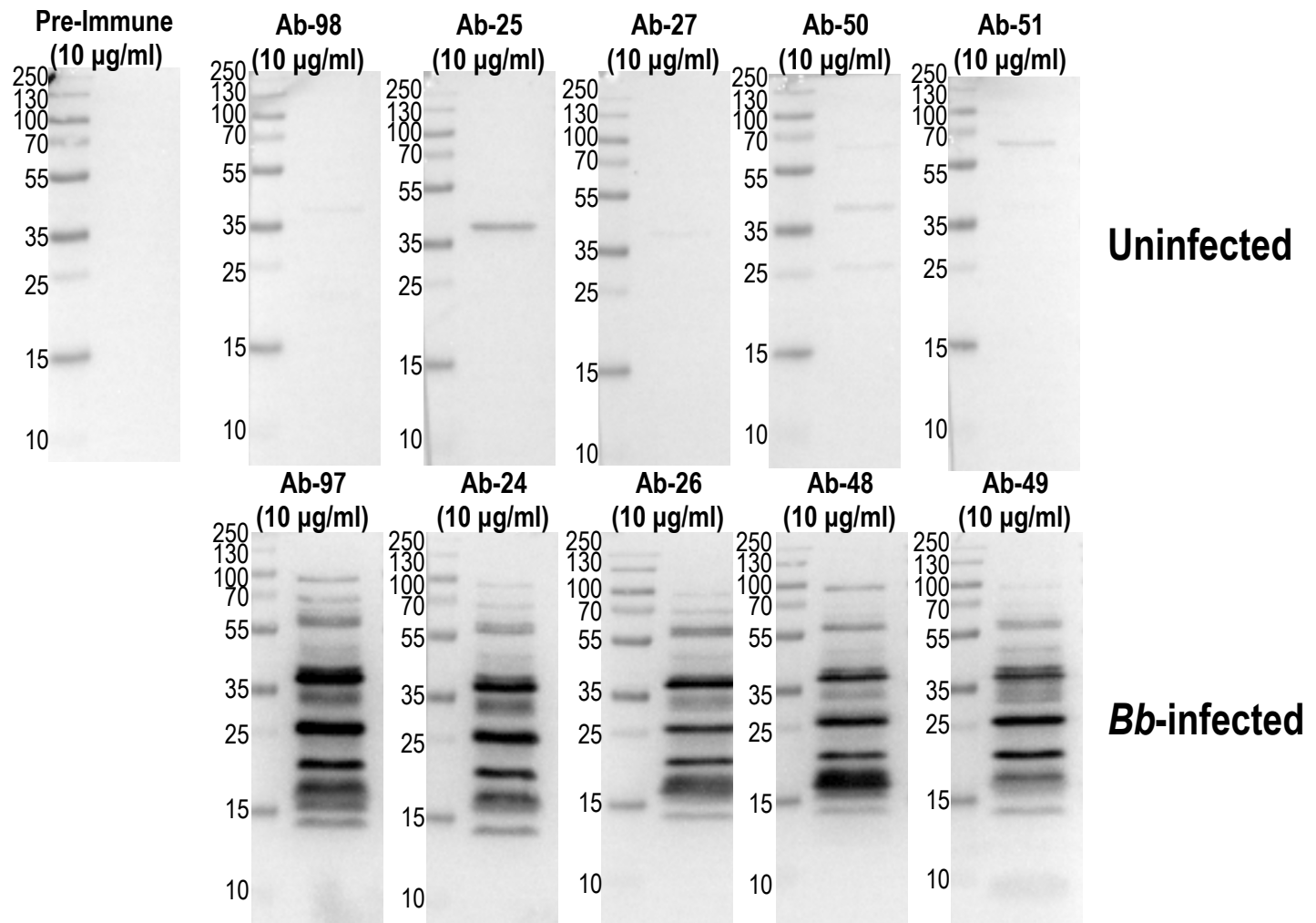

Supplement: Supplementary file 1 — Additional file 1: SF1. Non-invasive method of collecting saliva from Ixodes scapularis nymphs. Tick saliva collections were performed using a 10μl pipette tip set up. A modified 10μl pipette tip was used to affix the tick mouthpart in the solution and restrict the tick from escaping. Saliva collections from ticks were not included if leakage of fluid was detected around the protective cap. SF2. Antibody response to Borrelia burgdorferi antigens by ELISA and western blotting analyses. Total protein extracts from B. burgdorferi (1 or 3 μg) were coated per well for ELISA (A) or resolved by SDS-PAGE for western blotting (B) analyses using purified IgG (10μg/ ml) from pre-immune (PI), rabbit antibody (Ab) numbers 98, 25, 27, 50 and 51 from rabbits that were infested with uninfected nymphs and Ab numbers 97, 24, 26, 48, and 49 from rabbits that were infested with B. burgdorferi infected nymphs. For ELISA, the y-axis represents the A450 and x-axis represent the rabbit number. SF3. Profile of uninfected and Borrelia burgdorferi infected Ixodes scapularis nymph tick saliva proteins during feeding. Uninfected and B. burgdorferi infected I. scapularis nymph ticks that were unfed, partially fed for 12, 24, 36, 48, 60, and 72h, and replete-fed, were stimulated to salivate by injecting 2% pilocarpine into hemolymph. Saliva was electrophoresed on a 10-20% acrylamide gel and silver stained. Please note the molecular weight ladder from 10-250kDa. SF4. Secretion dynamics of all 747 proteins identified in uninfected and Borrelia burgdorferi infected Ixodes scapularis nymph tick saliva. Normalized spectral abundance factors (NSAF) values of all I. scapularis nymph tick saliva proteins identified in this study were normalized using the z-score statistics and then used to generate heat maps using heatmap2 function in gplots library using R as described in materials and methods. The red color represents high abundance to blue color indicating low abundance. SF5. Secretion dynamics of pro [file 12864_2021_7429_MOESM1_ESM.zip › SF2B_ESM.pdf]

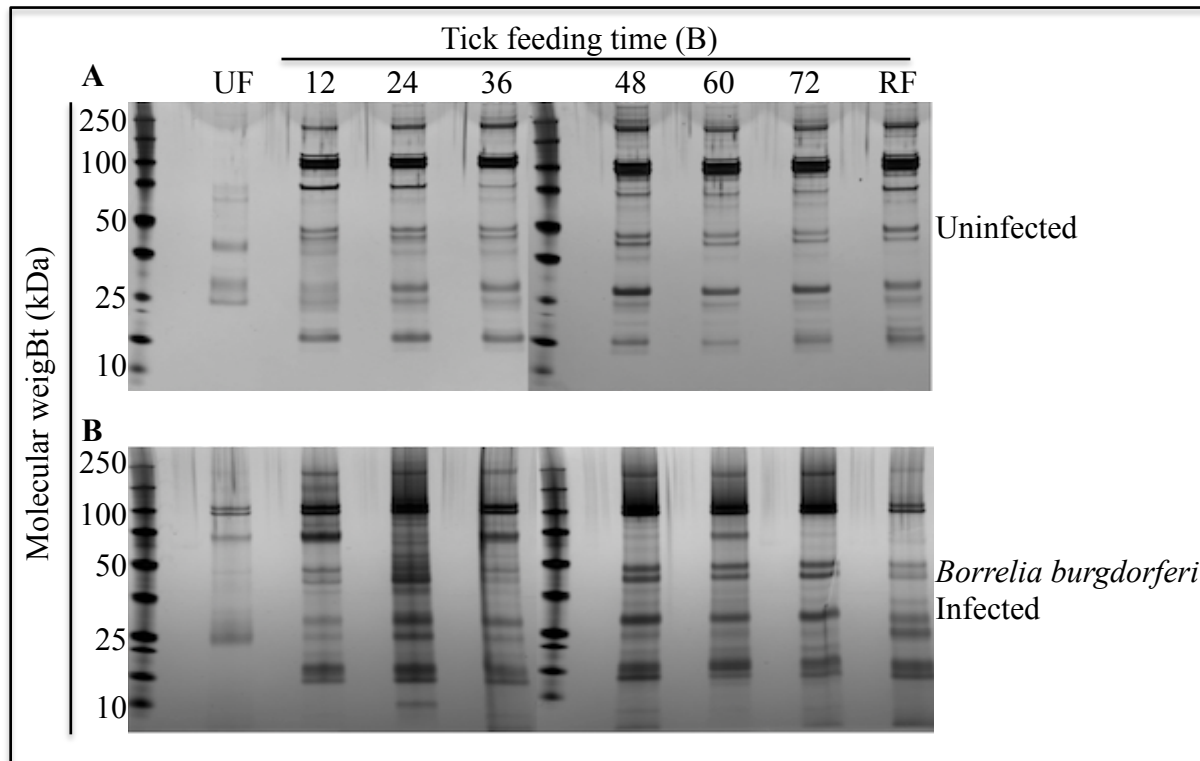

Supplemental figure 3

Supplement: Supplementary file 1 — Additional file 1: SF1. Non-invasive method of collecting saliva from Ixodes scapularis nymphs. Tick saliva collections were performed using a 10μl pipette tip set up. A modified 10μl pipette tip was used to affix the tick mouthpart in the solution and restrict the tick from escaping. Saliva collections from ticks were not included if leakage of fluid was detected around the protective cap. SF2. Antibody response to Borrelia burgdorferi antigens by ELISA and western blotting analyses. Total protein extracts from B. burgdorferi (1 or 3 μg) were coated per well for ELISA (A) or resolved by SDS-PAGE for western blotting (B) analyses using purified IgG (10μg/ ml) from pre-immune (PI), rabbit antibody (Ab) numbers 98, 25, 27, 50 and 51 from rabbits that were infested with uninfected nymphs and Ab numbers 97, 24, 26, 48, and 49 from rabbits that were infested with B. burgdorferi infected nymphs. For ELISA, the y-axis represents the A450 and x-axis represent the rabbit number. SF3. Profile of uninfected and Borrelia burgdorferi infected Ixodes scapularis nymph tick saliva proteins during feeding. Uninfected and B. burgdorferi infected I. scapularis nymph ticks that were unfed, partially fed for 12, 24, 36, 48, 60, and 72h, and replete-fed, were stimulated to salivate by injecting 2% pilocarpine into hemolymph. Saliva was electrophoresed on a 10-20% acrylamide gel and silver stained. Please note the molecular weight ladder from 10-250kDa. SF4. Secretion dynamics of all 747 proteins identified in uninfected and Borrelia burgdorferi infected Ixodes scapularis nymph tick saliva. Normalized spectral abundance factors (NSAF) values of all I. scapularis nymph tick saliva proteins identified in this study were normalized using the z-score statistics and then used to generate heat maps using heatmap2 function in gplots library using R as described in materials and methods. The red color represents high abundance to blue color indicating low abundance. SF5. Secretion dynamics of pro [file 12864_2021_7429_MOESM1_ESM.zip › SF3_ESM.pdf]

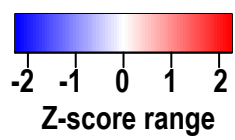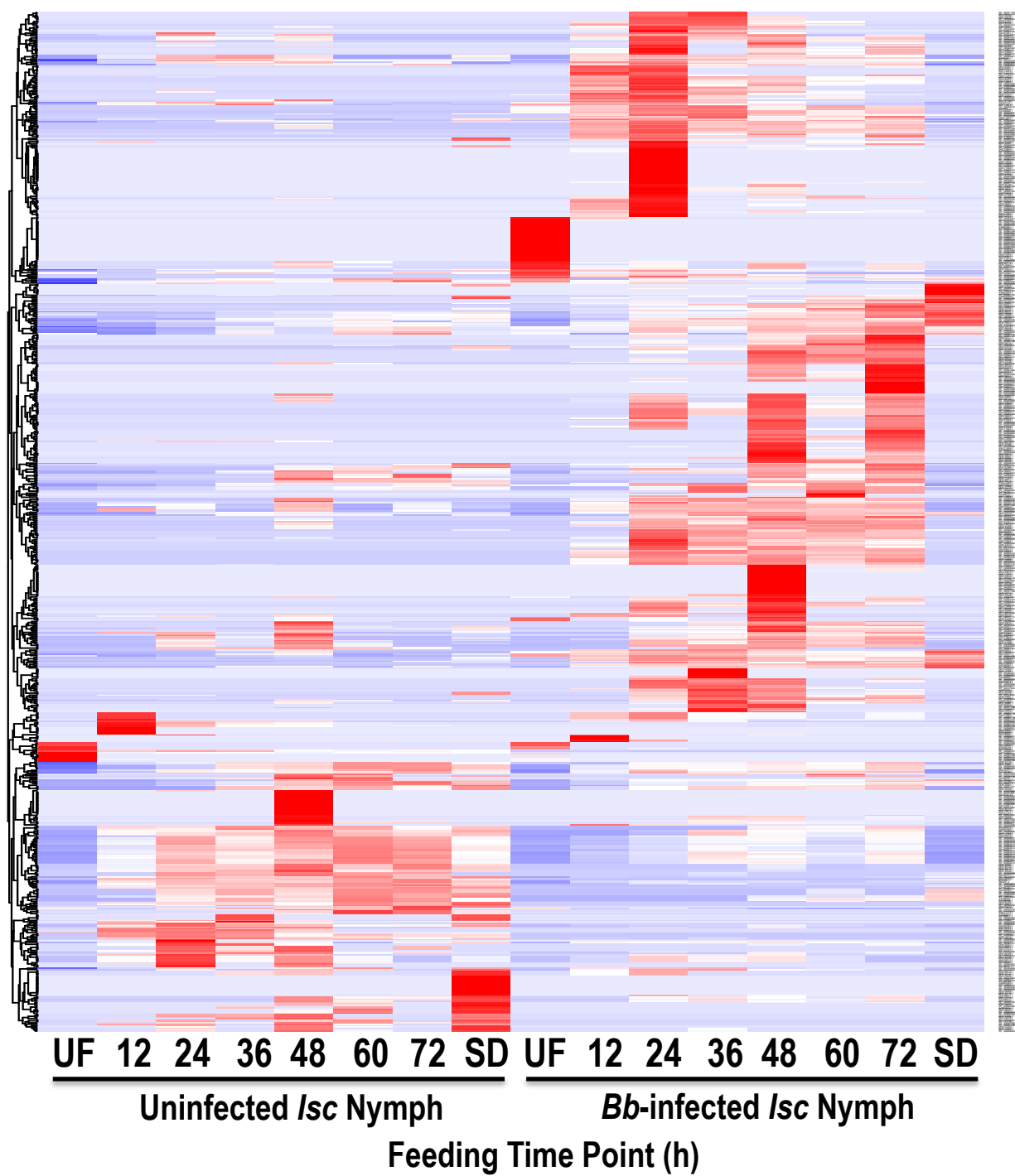

Supplemental figure 4

Supplement: Supplementary file 1 — Additional file 1: SF1. Non-invasive method of collecting saliva from Ixodes scapularis nymphs. Tick saliva collections were performed using a 10μl pipette tip set up. A modified 10μl pipette tip was used to affix the tick mouthpart in the solution and restrict the tick from escaping. Saliva collections from ticks were not included if leakage of fluid was detected around the protective cap. SF2. Antibody response to Borrelia burgdorferi antigens by ELISA and western blotting analyses. Total protein extracts from B. burgdorferi (1 or 3 μg) were coated per well for ELISA (A) or resolved by SDS-PAGE for western blotting (B) analyses using purified IgG (10μg/ ml) from pre-immune (PI), rabbit antibody (Ab) numbers 98, 25, 27, 50 and 51 from rabbits that were infested with uninfected nymphs and Ab numbers 97, 24, 26, 48, and 49 from rabbits that were infested with B. burgdorferi infected nymphs. For ELISA, the y-axis represents the A450 and x-axis represent the rabbit number. SF3. Profile of uninfected and Borrelia burgdorferi infected Ixodes scapularis nymph tick saliva proteins during feeding. Uninfected and B. burgdorferi infected I. scapularis nymph ticks that were unfed, partially fed for 12, 24, 36, 48, 60, and 72h, and replete-fed, were stimulated to salivate by injecting 2% pilocarpine into hemolymph. Saliva was electrophoresed on a 10-20% acrylamide gel and silver stained. Please note the molecular weight ladder from 10-250kDa. SF4. Secretion dynamics of all 747 proteins identified in uninfected and Borrelia burgdorferi infected Ixodes scapularis nymph tick saliva. Normalized spectral abundance factors (NSAF) values of all I. scapularis nymph tick saliva proteins identified in this study were normalized using the z-score statistics and then used to generate heat maps using heatmap2 function in gplots library using R as described in materials and methods. The red color represents high abundance to blue color indicating low abundance. SF5. Secretion dynamics of pro [file 12864_2021_7429_MOESM1_ESM.zip › SF4_ESM.pdf]

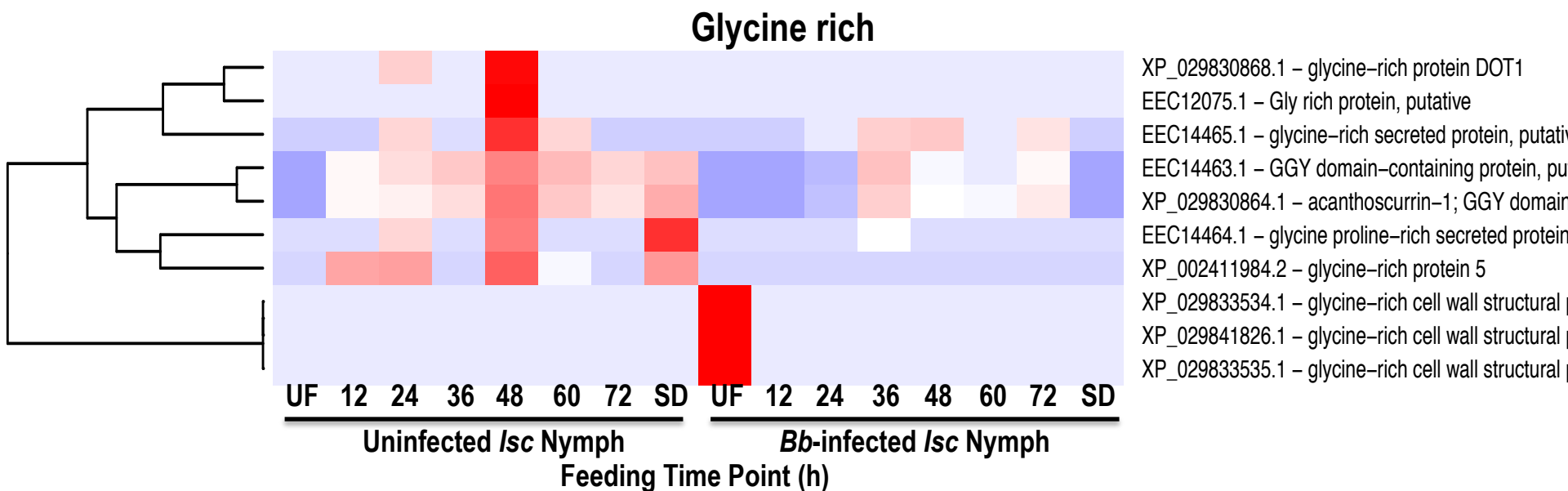

Supplement: Supplementary file 1 — Additional file 1: SF1. Non-invasive method of collecting saliva from Ixodes scapularis nymphs. Tick saliva collections were performed using a 10μl pipette tip set up. A modified 10μl pipette tip was used to affix the tick mouthpart in the solution and restrict the tick from escaping. Saliva collections from ticks were not included if leakage of fluid was detected around the protective cap. SF2. Antibody response to Borrelia burgdorferi antigens by ELISA and western blotting analyses. Total protein extracts from B. burgdorferi (1 or 3 μg) were coated per well for ELISA (A) or resolved by SDS-PAGE for western blotting (B) analyses using purified IgG (10μg/ ml) from pre-immune (PI), rabbit antibody (Ab) numbers 98, 25, 27, 50 and 51 from rabbits that were infested with uninfected nymphs and Ab numbers 97, 24, 26, 48, and 49 from rabbits that were infested with B. burgdorferi infected nymphs. For ELISA, the y-axis represents the A450 and x-axis represent the rabbit number. SF3. Profile of uninfected and Borrelia burgdorferi infected Ixodes scapularis nymph tick saliva proteins during feeding. Uninfected and B. burgdorferi infected I. scapularis nymph ticks that were unfed, partially fed for 12, 24, 36, 48, 60, and 72h, and replete-fed, were stimulated to salivate by injecting 2% pilocarpine into hemolymph. Saliva was electrophoresed on a 10-20% acrylamide gel and silver stained. Please note the molecular weight ladder from 10-250kDa. SF4. Secretion dynamics of all 747 proteins identified in uninfected and Borrelia burgdorferi infected Ixodes scapularis nymph tick saliva. Normalized spectral abundance factors (NSAF) values of all I. scapularis nymph tick saliva proteins identified in this study were normalized using the z-score statistics and then used to generate heat maps using heatmap2 function in gplots library using R as described in materials and methods. The red color represents high abundance to blue color indicating low abundance. SF5. Secretion dynamics of pro [file 12864_2021_7429_MOESM1_ESM.zip › SF5B_ESM.pdf]

# Cytoskeletal

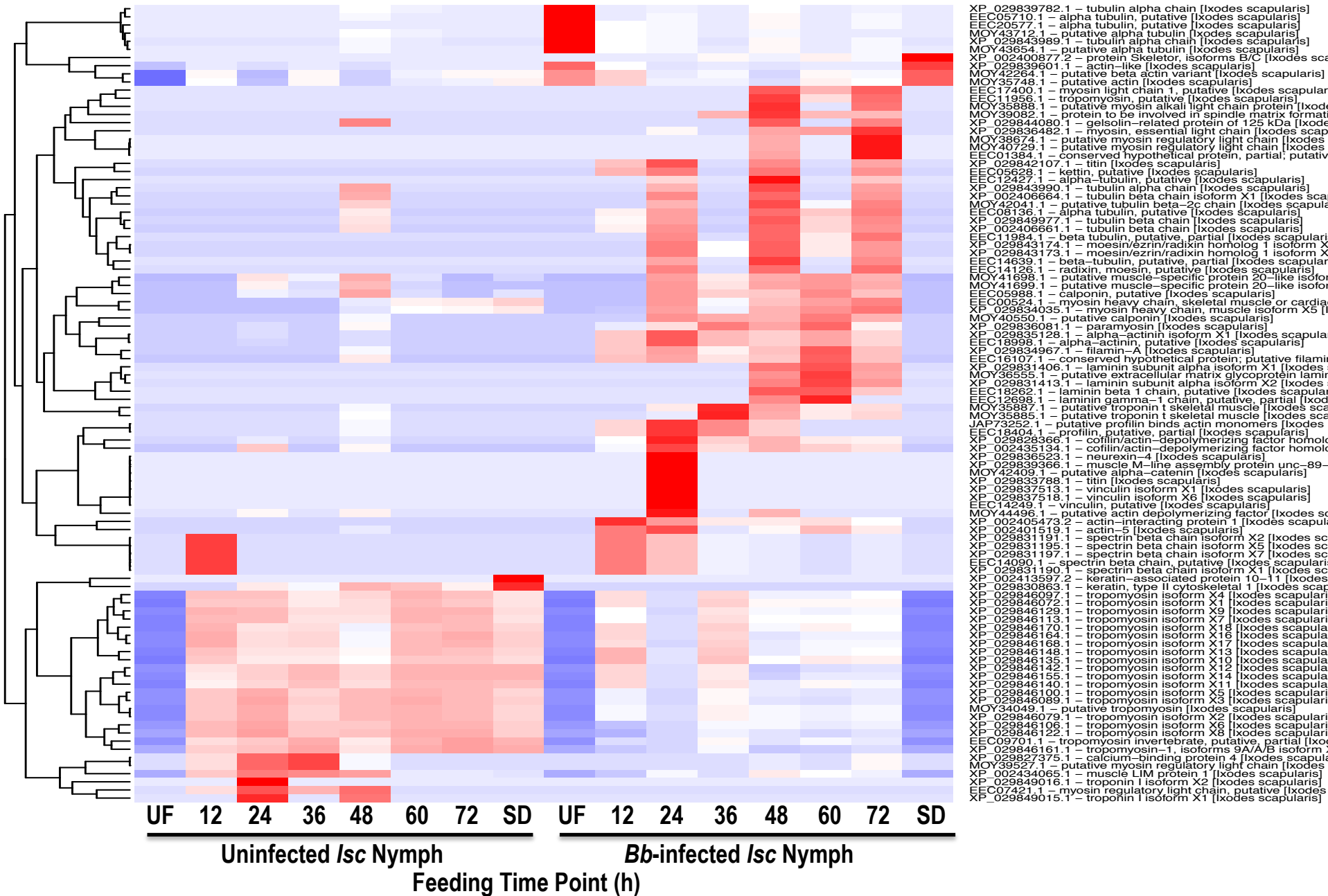

Supplemental figure 5D

Supplement: Supplementary file 1 — Additional file 1: SF1. Non-invasive method of collecting saliva from Ixodes scapularis nymphs. Tick saliva collections were performed using a 10μl pipette tip set up. A modified 10μl pipette tip was used to affix the tick mouthpart in the solution and restrict the tick from escaping. Saliva collections from ticks were not included if leakage of fluid was detected around the protective cap. SF2. Antibody response to Borrelia burgdorferi antigens by ELISA and western blotting analyses. Total protein extracts from B. burgdorferi (1 or 3 μg) were coated per well for ELISA (A) or resolved by SDS-PAGE for western blotting (B) analyses using purified IgG (10μg/ ml) from pre-immune (PI), rabbit antibody (Ab) numbers 98, 25, 27, 50 and 51 from rabbits that were infested with uninfected nymphs and Ab numbers 97, 24, 26, 48, and 49 from rabbits that were infested with B. burgdorferi infected nymphs. For ELISA, the y-axis represents the A450 and x-axis represent the rabbit number. SF3. Profile of uninfected and Borrelia burgdorferi infected Ixodes scapularis nymph tick saliva proteins during feeding. Uninfected and B. burgdorferi infected I. scapularis nymph ticks that were unfed, partially fed for 12, 24, 36, 48, 60, and 72h, and replete-fed, were stimulated to salivate by injecting 2% pilocarpine into hemolymph. Saliva was electrophoresed on a 10-20% acrylamide gel and silver stained. Please note the molecular weight ladder from 10-250kDa. SF4. Secretion dynamics of all 747 proteins identified in uninfected and Borrelia burgdorferi infected Ixodes scapularis nymph tick saliva. Normalized spectral abundance factors (NSAF) values of all I. scapularis nymph tick saliva proteins identified in this study were normalized using the z-score statistics and then used to generate heat maps using heatmap2 function in gplots library using R as described in materials and methods. The red color represents high abundance to blue color indicating low abundance. SF5. Secretion dynamics of pro [file 12864_2021_7429_MOESM1_ESM.zip › SF5D_ESM.pdf]

## Detoxification/ Antioxidant

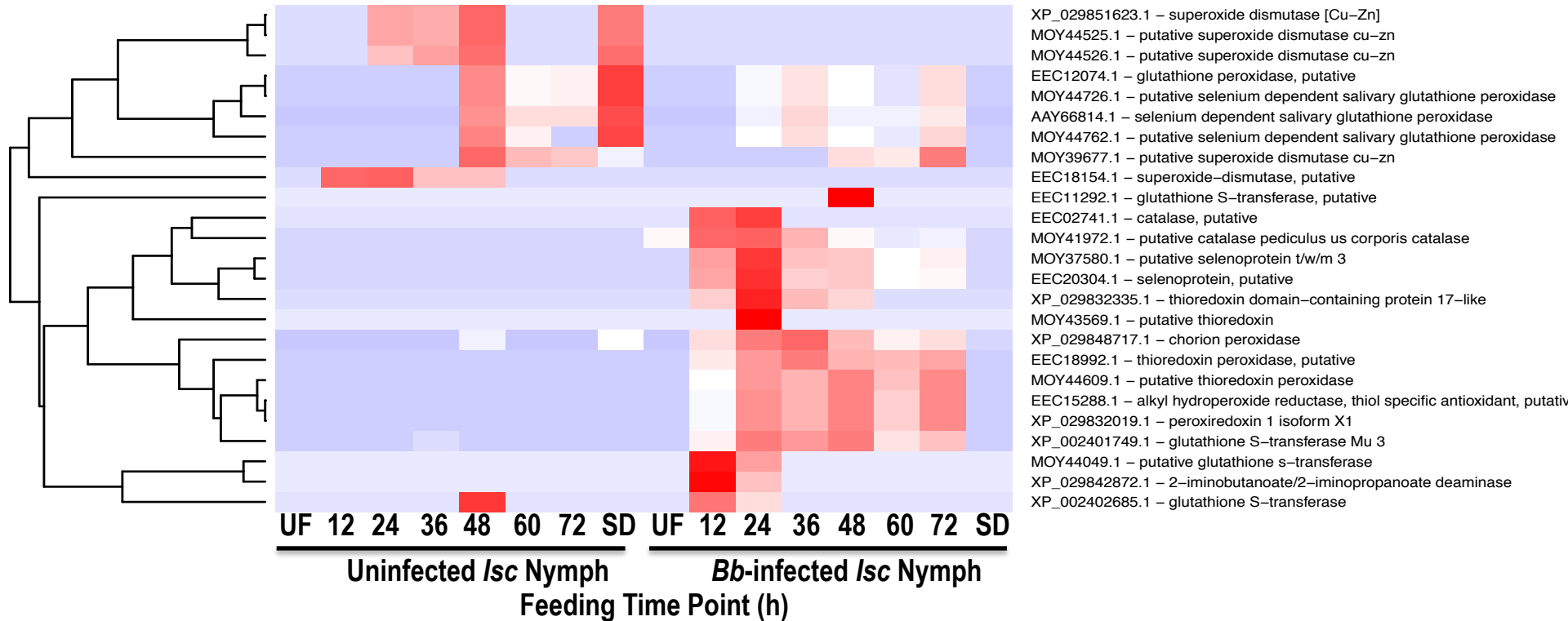

Supplement: Supplementary file 1 — Additional file 1: SF1. Non-invasive method of collecting saliva from Ixodes scapularis nymphs. Tick saliva collections were performed using a 10μl pipette tip set up. A modified 10μl pipette tip was used to affix the tick mouthpart in the solution and restrict the tick from escaping. Saliva collections from ticks were not included if leakage of fluid was detected around the protective cap. SF2. Antibody response to Borrelia burgdorferi antigens by ELISA and western blotting analyses. Total protein extracts from B. burgdorferi (1 or 3 μg) were coated per well for ELISA (A) or resolved by SDS-PAGE for western blotting (B) analyses using purified IgG (10μg/ ml) from pre-immune (PI), rabbit antibody (Ab) numbers 98, 25, 27, 50 and 51 from rabbits that were infested with uninfected nymphs and Ab numbers 97, 24, 26, 48, and 49 from rabbits that were infested with B. burgdorferi infected nymphs. For ELISA, the y-axis represents the A450 and x-axis represent the rabbit number. SF3. Profile of uninfected and Borrelia burgdorferi infected Ixodes scapularis nymph tick saliva proteins during feeding. Uninfected and B. burgdorferi infected I. scapularis nymph ticks that were unfed, partially fed for 12, 24, 36, 48, 60, and 72h, and replete-fed, were stimulated to salivate by injecting 2% pilocarpine into hemolymph. Saliva was electrophoresed on a 10-20% acrylamide gel and silver stained. Please note the molecular weight ladder from 10-250kDa. SF4. Secretion dynamics of all 747 proteins identified in uninfected and Borrelia burgdorferi infected Ixodes scapularis nymph tick saliva. Normalized spectral abundance factors (NSAF) values of all I. scapularis nymph tick saliva proteins identified in this study were normalized using the z-score statistics and then used to generate heat maps using heatmap2 function in gplots library using R as described in materials and methods. The red color represents high abundance to blue color indicating low abundance. SF5. Secretion dynamics of pro [file 12864_2021_7429_MOESM1_ESM.zip › SF5E_ESM.pdf]

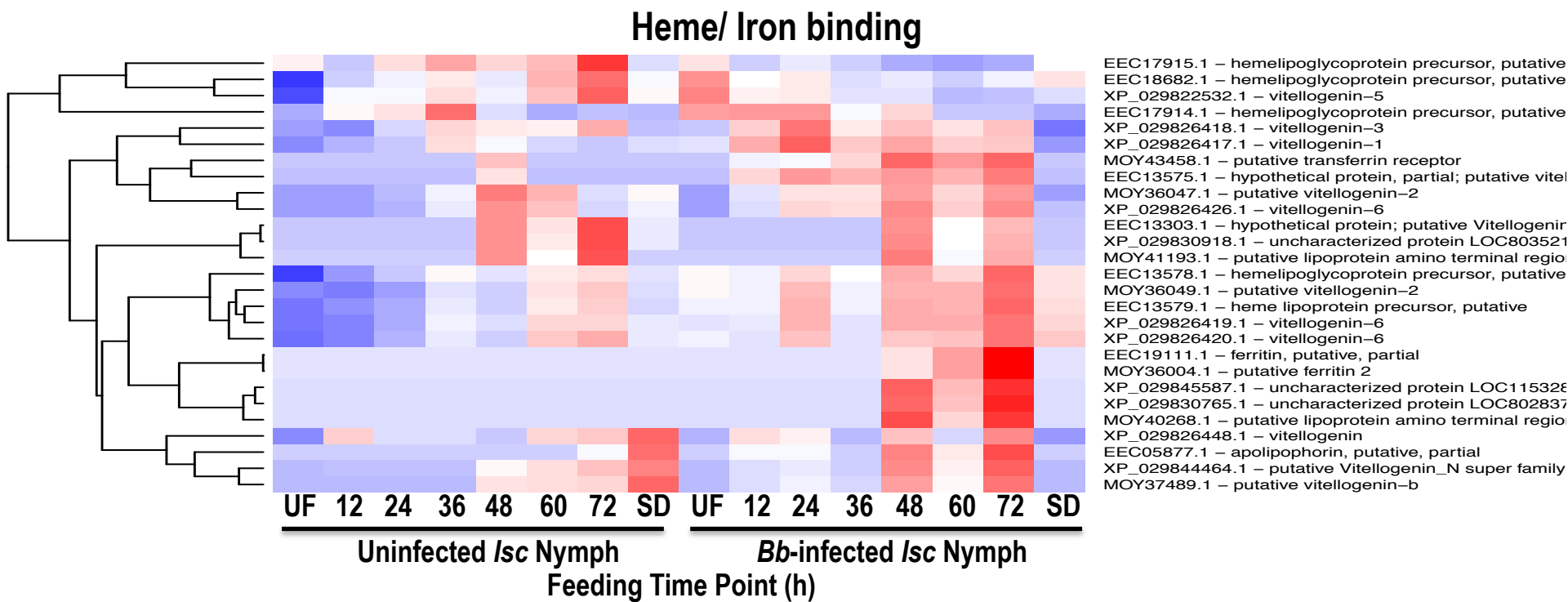

Supplement: Supplementary file 1 — Additional file 1: SF1. Non-invasive method of collecting saliva from Ixodes scapularis nymphs. Tick saliva collections were performed using a 10μl pipette tip set up. A modified 10μl pipette tip was used to affix the tick mouthpart in the solution and restrict the tick from escaping. Saliva collections from ticks were not included if leakage of fluid was detected around the protective cap. SF2. Antibody response to Borrelia burgdorferi antigens by ELISA and western blotting analyses. Total protein extracts from B. burgdorferi (1 or 3 μg) were coated per well for ELISA (A) or resolved by SDS-PAGE for western blotting (B) analyses using purified IgG (10μg/ ml) from pre-immune (PI), rabbit antibody (Ab) numbers 98, 25, 27, 50 and 51 from rabbits that were infested with uninfected nymphs and Ab numbers 97, 24, 26, 48, and 49 from rabbits that were infested with B. burgdorferi infected nymphs. For ELISA, the y-axis represents the A450 and x-axis represent the rabbit number. SF3. Profile of uninfected and Borrelia burgdorferi infected Ixodes scapularis nymph tick saliva proteins during feeding. Uninfected and B. burgdorferi infected I. scapularis nymph ticks that were unfed, partially fed for 12, 24, 36, 48, 60, and 72h, and replete-fed, were stimulated to salivate by injecting 2% pilocarpine into hemolymph. Saliva was electrophoresed on a 10-20% acrylamide gel and silver stained. Please note the molecular weight ladder from 10-250kDa. SF4. Secretion dynamics of all 747 proteins identified in uninfected and Borrelia burgdorferi infected Ixodes scapularis nymph tick saliva. Normalized spectral abundance factors (NSAF) values of all I. scapularis nymph tick saliva proteins identified in this study were normalized using the z-score statistics and then used to generate heat maps using heatmap2 function in gplots library using R as described in materials and methods. The red color represents high abundance to blue color indicating low abundance. SF5. Secretion dynamics of pro [file 12864_2021_7429_MOESM1_ESM.zip › SF5F_ESM.pdf]

Nuclear regulation

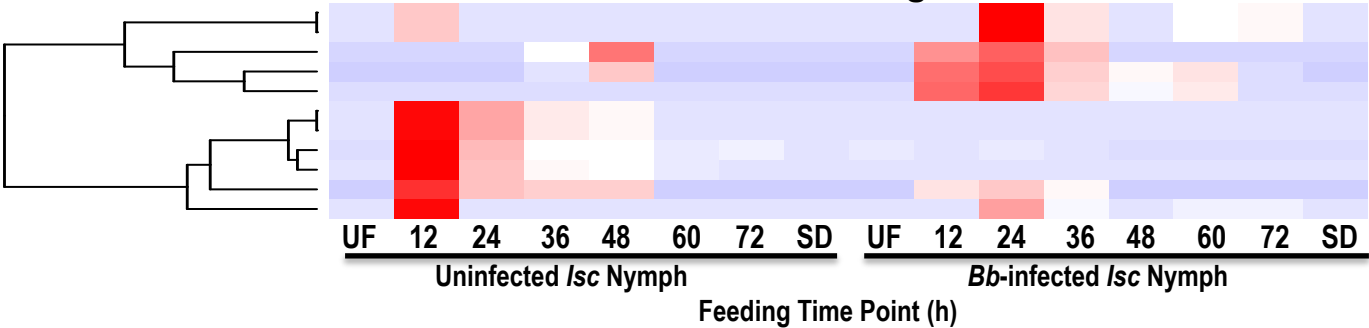

Supplement: Supplementary file 1 — Additional file 1: SF1. Non-invasive method of collecting saliva from Ixodes scapularis nymphs. Tick saliva collections were performed using a 10μl pipette tip set up. A modified 10μl pipette tip was used to affix the tick mouthpart in the solution and restrict the tick from escaping. Saliva collections from ticks were not included if leakage of fluid was detected around the protective cap. SF2. Antibody response to Borrelia burgdorferi antigens by ELISA and western blotting analyses. Total protein extracts from B. burgdorferi (1 or 3 μg) were coated per well for ELISA (A) or resolved by SDS-PAGE for western blotting (B) analyses using purified IgG (10μg/ ml) from pre-immune (PI), rabbit antibody (Ab) numbers 98, 25, 27, 50 and 51 from rabbits that were infested with uninfected nymphs and Ab numbers 97, 24, 26, 48, and 49 from rabbits that were infested with B. burgdorferi infected nymphs. For ELISA, the y-axis represents the A450 and x-axis represent the rabbit number. SF3. Profile of uninfected and Borrelia burgdorferi infected Ixodes scapularis nymph tick saliva proteins during feeding. Uninfected and B. burgdorferi infected I. scapularis nymph ticks that were unfed, partially fed for 12, 24, 36, 48, 60, and 72h, and replete-fed, were stimulated to salivate by injecting 2% pilocarpine into hemolymph. Saliva was electrophoresed on a 10-20% acrylamide gel and silver stained. Please note the molecular weight ladder from 10-250kDa. SF4. Secretion dynamics of all 747 proteins identified in uninfected and Borrelia burgdorferi infected Ixodes scapularis nymph tick saliva. Normalized spectral abundance factors (NSAF) values of all I. scapularis nymph tick saliva proteins identified in this study were normalized using the z-score statistics and then used to generate heat maps using heatmap2 function in gplots library using R as described in materials and methods. The red color represents high abundance to blue color indicating low abundance. SF5. Secretion dynamics of pro [file 12864_2021_7429_MOESM1_ESM.zip › SF5H_ESM.pdf]

## Transcription machinery

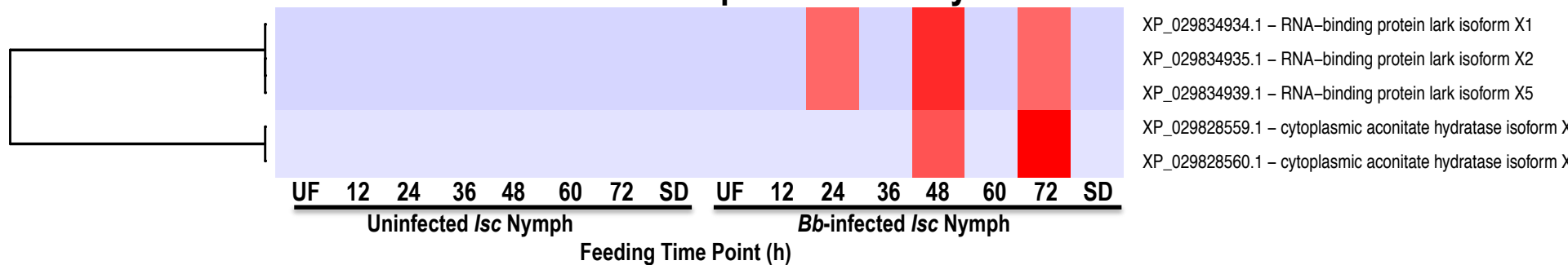

Supplement: Supplementary file 1 — Additional file 1: SF1. Non-invasive method of collecting saliva from Ixodes scapularis nymphs. Tick saliva collections were performed using a 10μl pipette tip set up. A modified 10μl pipette tip was used to affix the tick mouthpart in the solution and restrict the tick from escaping. Saliva collections from ticks were not included if leakage of fluid was detected around the protective cap. SF2. Antibody response to Borrelia burgdorferi antigens by ELISA and western blotting analyses. Total protein extracts from B. burgdorferi (1 or 3 μg) were coated per well for ELISA (A) or resolved by SDS-PAGE for western blotting (B) analyses using purified IgG (10μg/ ml) from pre-immune (PI), rabbit antibody (Ab) numbers 98, 25, 27, 50 and 51 from rabbits that were infested with uninfected nymphs and Ab numbers 97, 24, 26, 48, and 49 from rabbits that were infested with B. burgdorferi infected nymphs. For ELISA, the y-axis represents the A450 and x-axis represent the rabbit number. SF3. Profile of uninfected and Borrelia burgdorferi infected Ixodes scapularis nymph tick saliva proteins during feeding. Uninfected and B. burgdorferi infected I. scapularis nymph ticks that were unfed, partially fed for 12, 24, 36, 48, 60, and 72h, and replete-fed, were stimulated to salivate by injecting 2% pilocarpine into hemolymph. Saliva was electrophoresed on a 10-20% acrylamide gel and silver stained. Please note the molecular weight ladder from 10-250kDa. SF4. Secretion dynamics of all 747 proteins identified in uninfected and Borrelia burgdorferi infected Ixodes scapularis nymph tick saliva. Normalized spectral abundance factors (NSAF) values of all I. scapularis nymph tick saliva proteins identified in this study were normalized using the z-score statistics and then used to generate heat maps using heatmap2 function in gplots library using R as described in materials and methods. The red color represents high abundance to blue color indicating low abundance. SF5. Secretion dynamics of pro [file 12864_2021_7429_MOESM1_ESM.zip › SF5I_ESM.pdf]

# Metabolism, amino acids

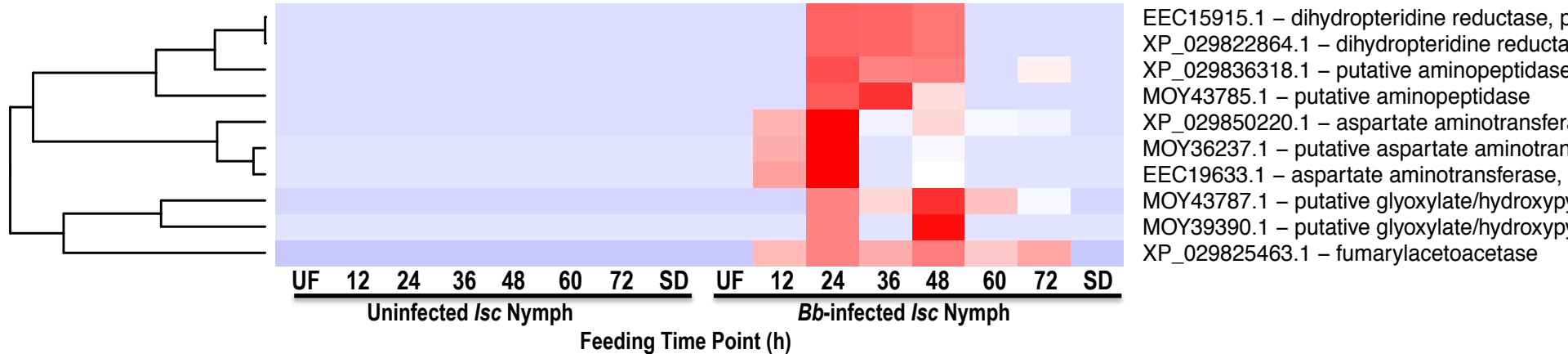

Supplement: Supplementary file 1 — Additional file 1: SF1. Non-invasive method of collecting saliva from Ixodes scapularis nymphs. Tick saliva collections were performed using a 10μl pipette tip set up. A modified 10μl pipette tip was used to affix the tick mouthpart in the solution and restrict the tick from escaping. Saliva collections from ticks were not included if leakage of fluid was detected around the protective cap. SF2. Antibody response to Borrelia burgdorferi antigens by ELISA and western blotting analyses. Total protein extracts from B. burgdorferi (1 or 3 μg) were coated per well for ELISA (A) or resolved by SDS-PAGE for western blotting (B) analyses using purified IgG (10μg/ ml) from pre-immune (PI), rabbit antibody (Ab) numbers 98, 25, 27, 50 and 51 from rabbits that were infested with uninfected nymphs and Ab numbers 97, 24, 26, 48, and 49 from rabbits that were infested with B. burgdorferi infected nymphs. For ELISA, the y-axis represents the A450 and x-axis represent the rabbit number. SF3. Profile of uninfected and Borrelia burgdorferi infected Ixodes scapularis nymph tick saliva proteins during feeding. Uninfected and B. burgdorferi infected I. scapularis nymph ticks that were unfed, partially fed for 12, 24, 36, 48, 60, and 72h, and replete-fed, were stimulated to salivate by injecting 2% pilocarpine into hemolymph. Saliva was electrophoresed on a 10-20% acrylamide gel and silver stained. Please note the molecular weight ladder from 10-250kDa. SF4. Secretion dynamics of all 747 proteins identified in uninfected and Borrelia burgdorferi infected Ixodes scapularis nymph tick saliva. Normalized spectral abundance factors (NSAF) values of all I. scapularis nymph tick saliva proteins identified in this study were normalized using the z-score statistics and then used to generate heat maps using heatmap2 function in gplots library using R as described in materials and methods. The red color represents high abundance to blue color indicating low abundance. SF5. Secretion dynamics of pro [file 12864_2021_7429_MOESM1_ESM.zip › SF5J_ESM.pdf]

## Metabolism, carbohydrates

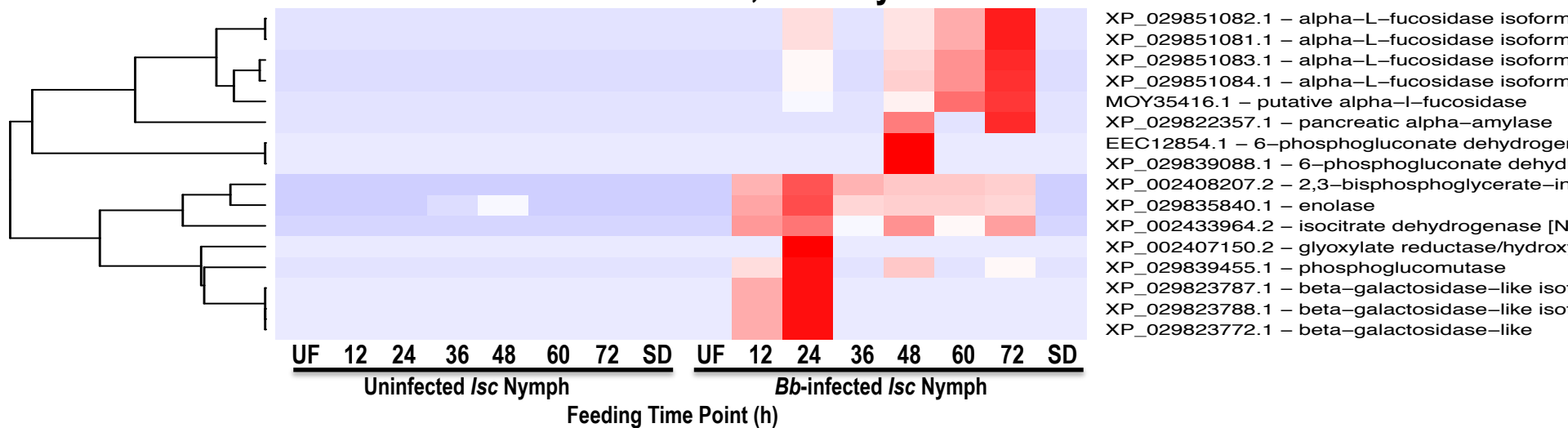

Supplement: Supplementary file 1 — Additional file 1: SF1. Non-invasive method of collecting saliva from Ixodes scapularis nymphs. Tick saliva collections were performed using a 10μl pipette tip set up. A modified 10μl pipette tip was used to affix the tick mouthpart in the solution and restrict the tick from escaping. Saliva collections from ticks were not included if leakage of fluid was detected around the protective cap. SF2. Antibody response to Borrelia burgdorferi antigens by ELISA and western blotting analyses. Total protein extracts from B. burgdorferi (1 or 3 μg) were coated per well for ELISA (A) or resolved by SDS-PAGE for western blotting (B) analyses using purified IgG (10μg/ ml) from pre-immune (PI), rabbit antibody (Ab) numbers 98, 25, 27, 50 and 51 from rabbits that were infested with uninfected nymphs and Ab numbers 97, 24, 26, 48, and 49 from rabbits that were infested with B. burgdorferi infected nymphs. For ELISA, the y-axis represents the A450 and x-axis represent the rabbit number. SF3. Profile of uninfected and Borrelia burgdorferi infected Ixodes scapularis nymph tick saliva proteins during feeding. Uninfected and B. burgdorferi infected I. scapularis nymph ticks that were unfed, partially fed for 12, 24, 36, 48, 60, and 72h, and replete-fed, were stimulated to salivate by injecting 2% pilocarpine into hemolymph. Saliva was electrophoresed on a 10-20% acrylamide gel and silver stained. Please note the molecular weight ladder from 10-250kDa. SF4. Secretion dynamics of all 747 proteins identified in uninfected and Borrelia burgdorferi infected Ixodes scapularis nymph tick saliva. Normalized spectral abundance factors (NSAF) values of all I. scapularis nymph tick saliva proteins identified in this study were normalized using the z-score statistics and then used to generate heat maps using heatmap2 function in gplots library using R as described in materials and methods. The red color represents high abundance to blue color indicating low abundance. SF5. Secretion dynamics of pro [file 12864_2021_7429_MOESM1_ESM.zip › SF5K_ESM.pdf]

## Protein modification

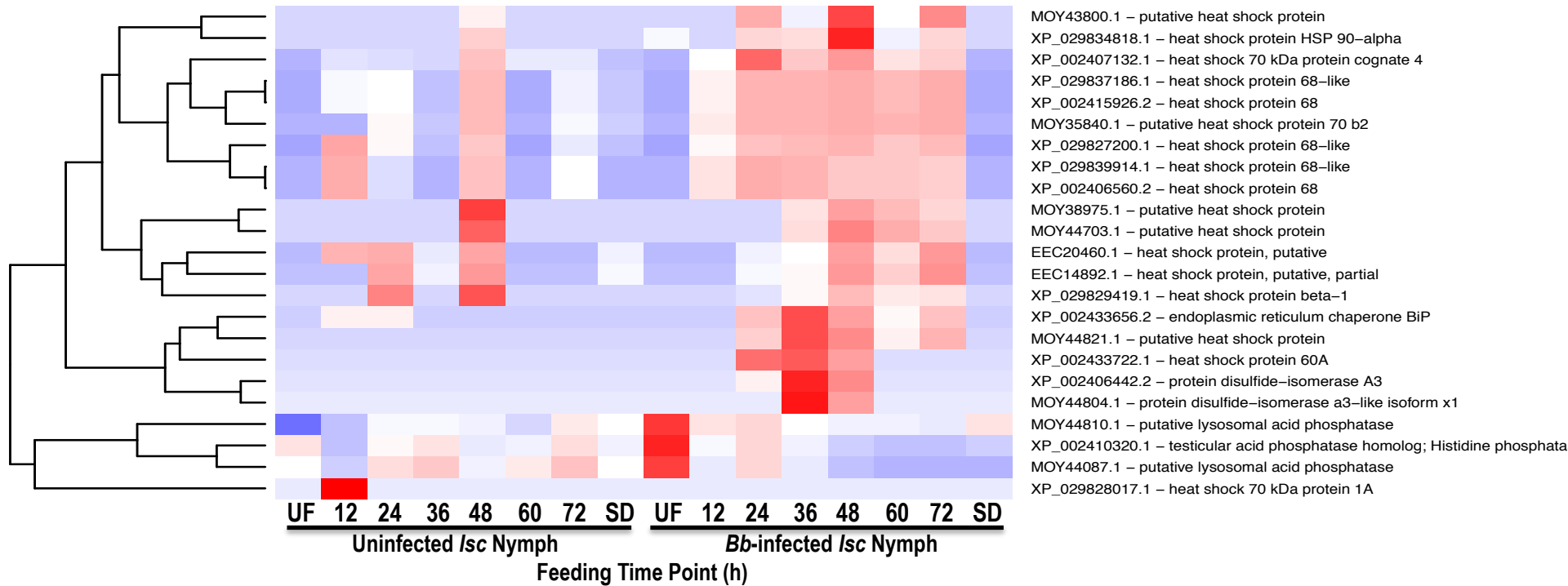

Supplement: Supplementary file 1 — Additional file 1: SF1. Non-invasive method of collecting saliva from Ixodes scapularis nymphs. Tick saliva collections were performed using a 10μl pipette tip set up. A modified 10μl pipette tip was used to affix the tick mouthpart in the solution and restrict the tick from escaping. Saliva collections from ticks were not included if leakage of fluid was detected around the protective cap. SF2. Antibody response to Borrelia burgdorferi antigens by ELISA and western blotting analyses. Total protein extracts from B. burgdorferi (1 or 3 μg) were coated per well for ELISA (A) or resolved by SDS-PAGE for western blotting (B) analyses using purified IgG (10μg/ ml) from pre-immune (PI), rabbit antibody (Ab) numbers 98, 25, 27, 50 and 51 from rabbits that were infested with uninfected nymphs and Ab numbers 97, 24, 26, 48, and 49 from rabbits that were infested with B. burgdorferi infected nymphs. For ELISA, the y-axis represents the A450 and x-axis represent the rabbit number. SF3. Profile of uninfected and Borrelia burgdorferi infected Ixodes scapularis nymph tick saliva proteins during feeding. Uninfected and B. burgdorferi infected I. scapularis nymph ticks that were unfed, partially fed for 12, 24, 36, 48, 60, and 72h, and replete-fed, were stimulated to salivate by injecting 2% pilocarpine into hemolymph. Saliva was electrophoresed on a 10-20% acrylamide gel and silver stained. Please note the molecular weight ladder from 10-250kDa. SF4. Secretion dynamics of all 747 proteins identified in uninfected and Borrelia burgdorferi infected Ixodes scapularis nymph tick saliva. Normalized spectral abundance factors (NSAF) values of all I. scapularis nymph tick saliva proteins identified in this study were normalized using the z-score statistics and then used to generate heat maps using heatmap2 function in gplots library using R as described in materials and methods. The red color represents high abundance to blue color indicating low abundance. SF5. Secretion dynamics of pro [file 12864_2021_7429_MOESM1_ESM.zip › SF5M_ESM.pdf]

Protein synthesis

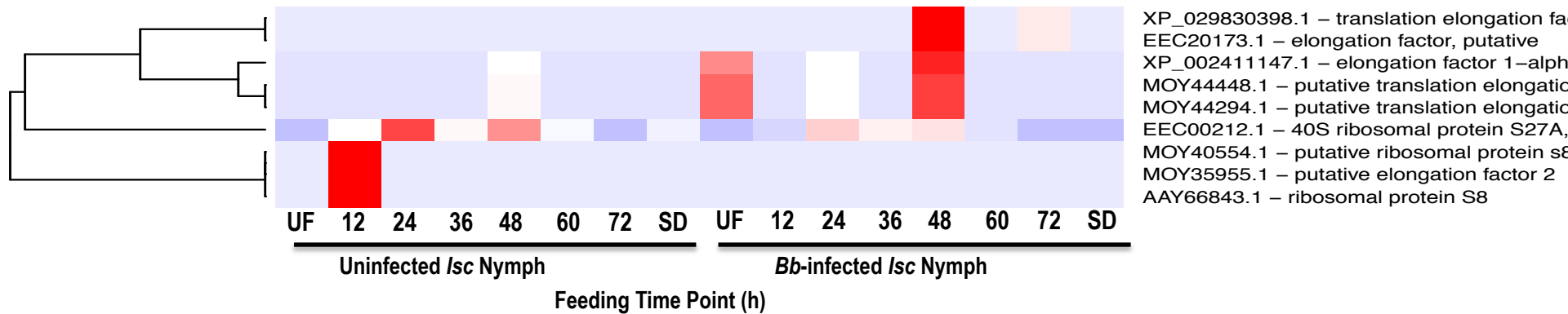

Supplemental figure 50

Supplement: Supplementary file 1 — Additional file 1: SF1. Non-invasive method of collecting saliva from Ixodes scapularis nymphs. Tick saliva collections were performed using a 10μl pipette tip set up. A modified 10μl pipette tip was used to affix the tick mouthpart in the solution and restrict the tick from escaping. Saliva collections from ticks were not included if leakage of fluid was detected around the protective cap. SF2. Antibody response to Borrelia burgdorferi antigens by ELISA and western blotting analyses. Total protein extracts from B. burgdorferi (1 or 3 μg) were coated per well for ELISA (A) or resolved by SDS-PAGE for western blotting (B) analyses using purified IgG (10μg/ ml) from pre-immune (PI), rabbit antibody (Ab) numbers 98, 25, 27, 50 and 51 from rabbits that were infested with uninfected nymphs and Ab numbers 97, 24, 26, 48, and 49 from rabbits that were infested with B. burgdorferi infected nymphs. For ELISA, the y-axis represents the A450 and x-axis represent the rabbit number. SF3. Profile of uninfected and Borrelia burgdorferi infected Ixodes scapularis nymph tick saliva proteins during feeding. Uninfected and B. burgdorferi infected I. scapularis nymph ticks that were unfed, partially fed for 12, 24, 36, 48, 60, and 72h, and replete-fed, were stimulated to salivate by injecting 2% pilocarpine into hemolymph. Saliva was electrophoresed on a 10-20% acrylamide gel and silver stained. Please note the molecular weight ladder from 10-250kDa. SF4. Secretion dynamics of all 747 proteins identified in uninfected and Borrelia burgdorferi infected Ixodes scapularis nymph tick saliva. Normalized spectral abundance factors (NSAF) values of all I. scapularis nymph tick saliva proteins identified in this study were normalized using the z-score statistics and then used to generate heat maps using heatmap2 function in gplots library using R as described in materials and methods. The red color represents high abundance to blue color indicating low abundance. SF5. Secretion dynamics of pro [file 12864_2021_7429_MOESM1_ESM.zip › SF5O_ESM.pdf]

## Transporters/ Receptors

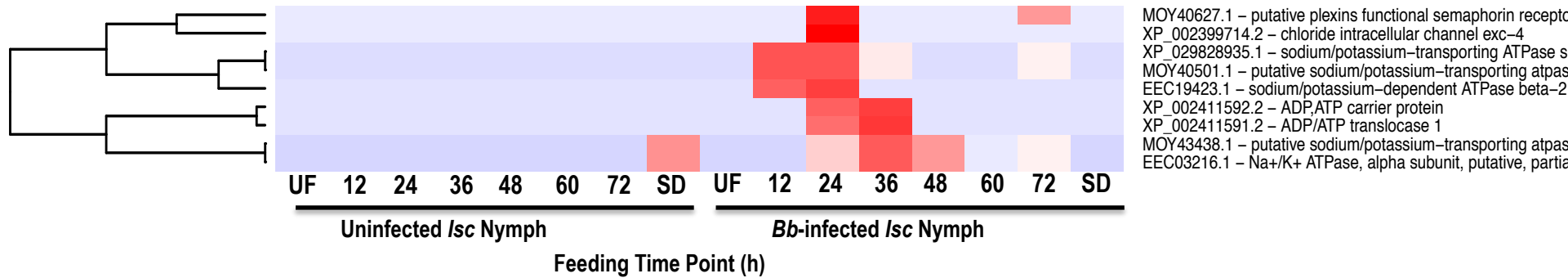

Supplement: Supplementary file 1 — Additional file 1: SF1. Non-invasive method of collecting saliva from Ixodes scapularis nymphs. Tick saliva collections were performed using a 10μl pipette tip set up. A modified 10μl pipette tip was used to affix the tick mouthpart in the solution and restrict the tick from escaping. Saliva collections from ticks were not included if leakage of fluid was detected around the protective cap. SF2. Antibody response to Borrelia burgdorferi antigens by ELISA and western blotting analyses. Total protein extracts from B. burgdorferi (1 or 3 μg) were coated per well for ELISA (A) or resolved by SDS-PAGE for western blotting (B) analyses using purified IgG (10μg/ ml) from pre-immune (PI), rabbit antibody (Ab) numbers 98, 25, 27, 50 and 51 from rabbits that were infested with uninfected nymphs and Ab numbers 97, 24, 26, 48, and 49 from rabbits that were infested with B. burgdorferi infected nymphs. For ELISA, the y-axis represents the A450 and x-axis represent the rabbit number. SF3. Profile of uninfected and Borrelia burgdorferi infected Ixodes scapularis nymph tick saliva proteins during feeding. Uninfected and B. burgdorferi infected I. scapularis nymph ticks that were unfed, partially fed for 12, 24, 36, 48, 60, and 72h, and replete-fed, were stimulated to salivate by injecting 2% pilocarpine into hemolymph. Saliva was electrophoresed on a 10-20% acrylamide gel and silver stained. Please note the molecular weight ladder from 10-250kDa. SF4. Secretion dynamics of all 747 proteins identified in uninfected and Borrelia burgdorferi infected Ixodes scapularis nymph tick saliva. Normalized spectral abundance factors (NSAF) values of all I. scapularis nymph tick saliva proteins identified in this study were normalized using the z-score statistics and then used to generate heat maps using heatmap2 function in gplots library using R as described in materials and methods. The red color represents high abundance to blue color indicating low abundance. SF5. Secretion dynamics of pro [file 12864_2021_7429_MOESM1_ESM.zip › SF5Q_ESM.pdf]

## Signal Transduction

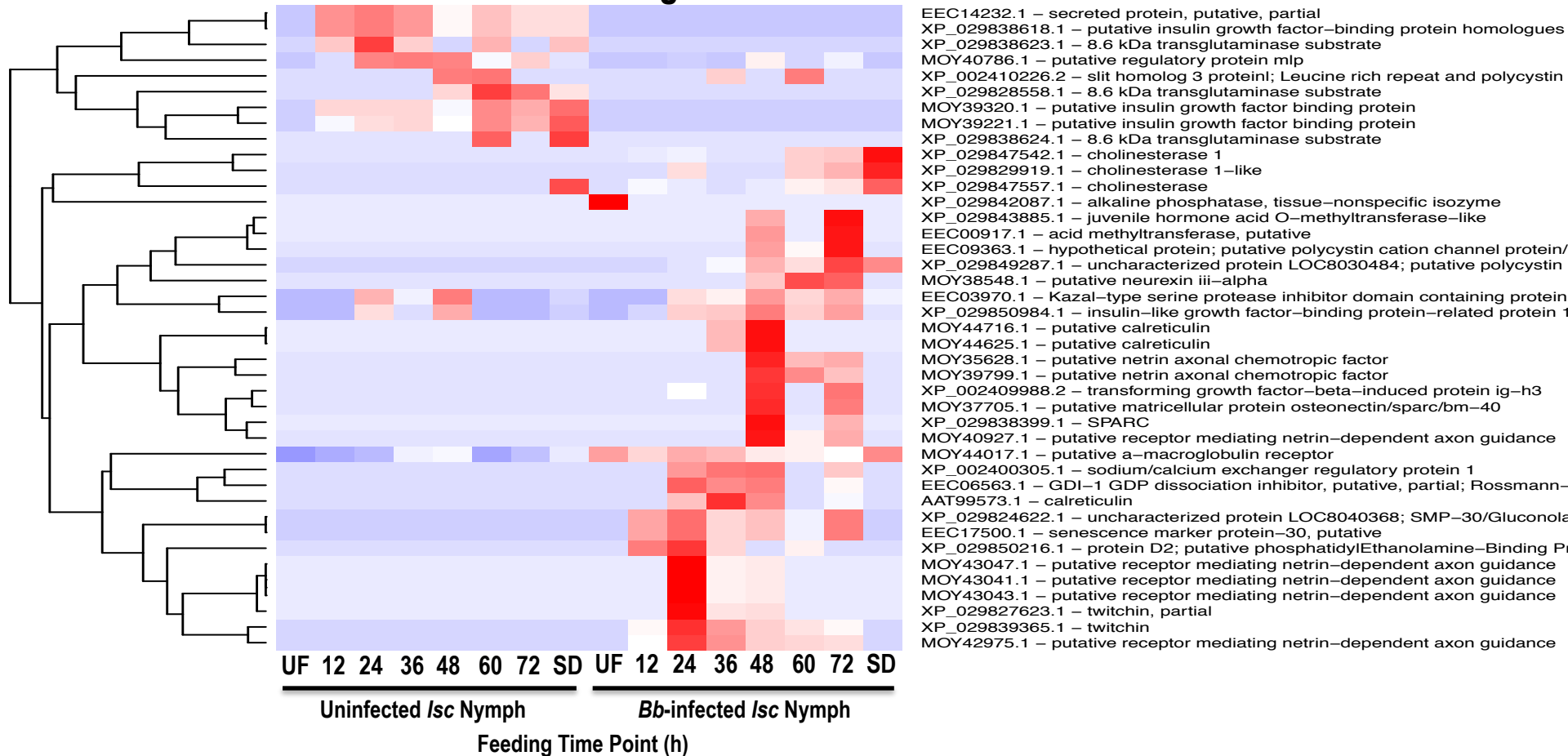

Supplement: Supplementary file 1 — Additional file 1: SF1. Non-invasive method of collecting saliva from Ixodes scapularis nymphs. Tick saliva collections were performed using a 10μl pipette tip set up. A modified 10μl pipette tip was used to affix the tick mouthpart in the solution and restrict the tick from escaping. Saliva collections from ticks were not included if leakage of fluid was detected around the protective cap. SF2. Antibody response to Borrelia burgdorferi antigens by ELISA and western blotting analyses. Total protein extracts from B. burgdorferi (1 or 3 μg) were coated per well for ELISA (A) or resolved by SDS-PAGE for western blotting (B) analyses using purified IgG (10μg/ ml) from pre-immune (PI), rabbit antibody (Ab) numbers 98, 25, 27, 50 and 51 from rabbits that were infested with uninfected nymphs and Ab numbers 97, 24, 26, 48, and 49 from rabbits that were infested with B. burgdorferi infected nymphs. For ELISA, the y-axis represents the A450 and x-axis represent the rabbit number. SF3. Profile of uninfected and Borrelia burgdorferi infected Ixodes scapularis nymph tick saliva proteins during feeding. Uninfected and B. burgdorferi infected I. scapularis nymph ticks that were unfed, partially fed for 12, 24, 36, 48, 60, and 72h, and replete-fed, were stimulated to salivate by injecting 2% pilocarpine into hemolymph. Saliva was electrophoresed on a 10-20% acrylamide gel and silver stained. Please note the molecular weight ladder from 10-250kDa. SF4. Secretion dynamics of all 747 proteins identified in uninfected and Borrelia burgdorferi infected Ixodes scapularis nymph tick saliva. Normalized spectral abundance factors (NSAF) values of all I. scapularis nymph tick saliva proteins identified in this study were normalized using the z-score statistics and then used to generate heat maps using heatmap2 function in gplots library using R as described in materials and methods. The red color represents high abundance to blue color indicating low abundance. SF5. Secretion dynamics of pro [file 12864_2021_7429_MOESM1_ESM.zip › SF5R_ESM.pdf]

# TSP

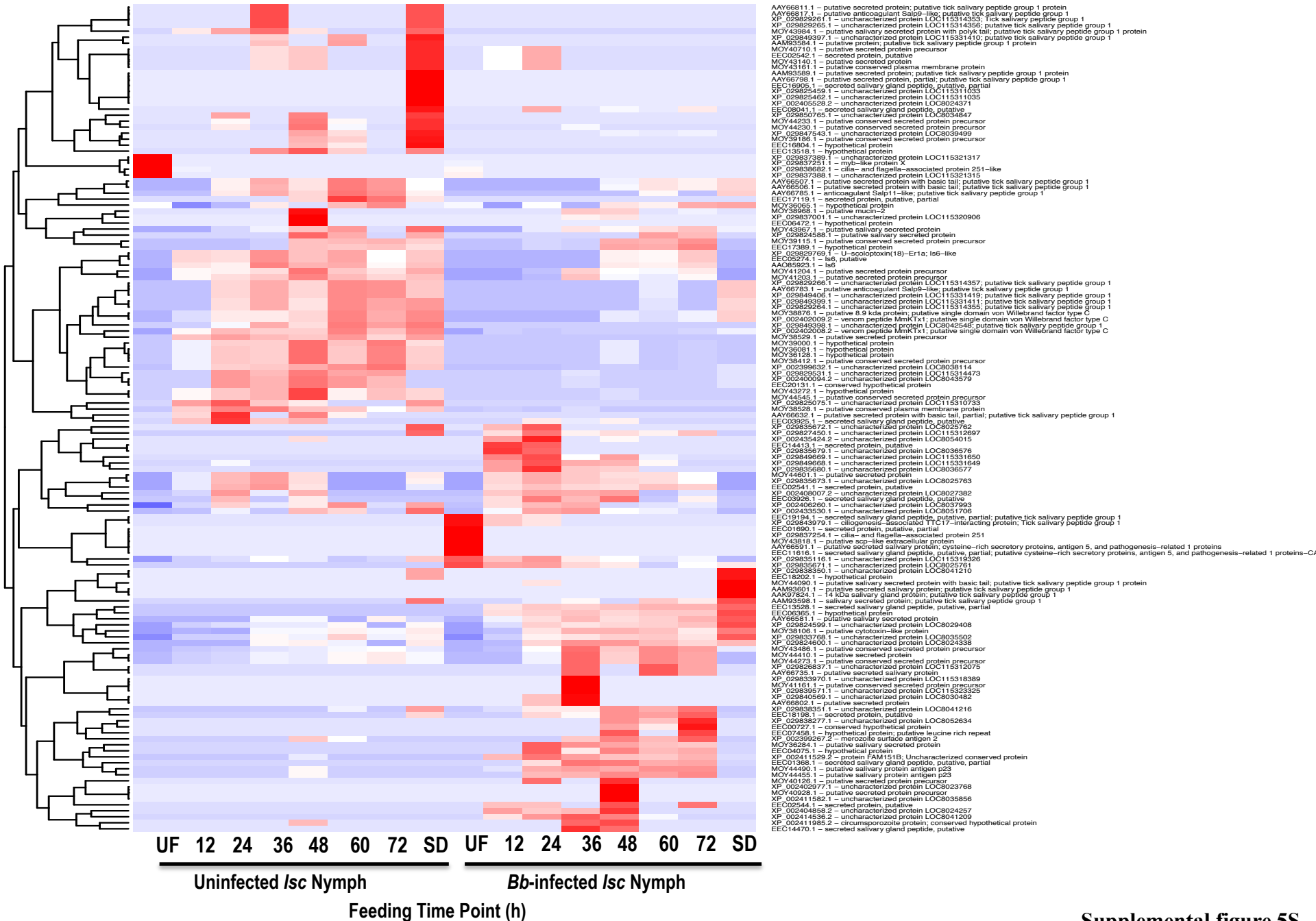

Supplement: Supplementary file 1 — Additional file 1: SF1. Non-invasive method of collecting saliva from Ixodes scapularis nymphs. Tick saliva collections were performed using a 10μl pipette tip set up. A modified 10μl pipette tip was used to affix the tick mouthpart in the solution and restrict the tick from escaping. Saliva collections from ticks were not included if leakage of fluid was detected around the protective cap. SF2. Antibody response to Borrelia burgdorferi antigens by ELISA and western blotting analyses. Total protein extracts from B. burgdorferi (1 or 3 μg) were coated per well for ELISA (A) or resolved by SDS-PAGE for western blotting (B) analyses using purified IgG (10μg/ ml) from pre-immune (PI), rabbit antibody (Ab) numbers 98, 25, 27, 50 and 51 from rabbits that were infested with uninfected nymphs and Ab numbers 97, 24, 26, 48, and 49 from rabbits that were infested with B. burgdorferi infected nymphs. For ELISA, the y-axis represents the A450 and x-axis represent the rabbit number. SF3. Profile of uninfected and Borrelia burgdorferi infected Ixodes scapularis nymph tick saliva proteins during feeding. Uninfected and B. burgdorferi infected I. scapularis nymph ticks that were unfed, partially fed for 12, 24, 36, 48, 60, and 72h, and replete-fed, were stimulated to salivate by injecting 2% pilocarpine into hemolymph. Saliva was electrophoresed on a 10-20% acrylamide gel and silver stained. Please note the molecular weight ladder from 10-250kDa. SF4. Secretion dynamics of all 747 proteins identified in uninfected and Borrelia burgdorferi infected Ixodes scapularis nymph tick saliva. Normalized spectral abundance factors (NSAF) values of all I. scapularis nymph tick saliva proteins identified in this study were normalized using the z-score statistics and then used to generate heat maps using heatmap2 function in gplots library using R as described in materials and methods. The red color represents high abundance to blue color indicating low abundance. SF5. Secretion dynamics of pro [file 12864_2021_7429_MOESM1_ESM.zip › SF5S_ESM.pdf]

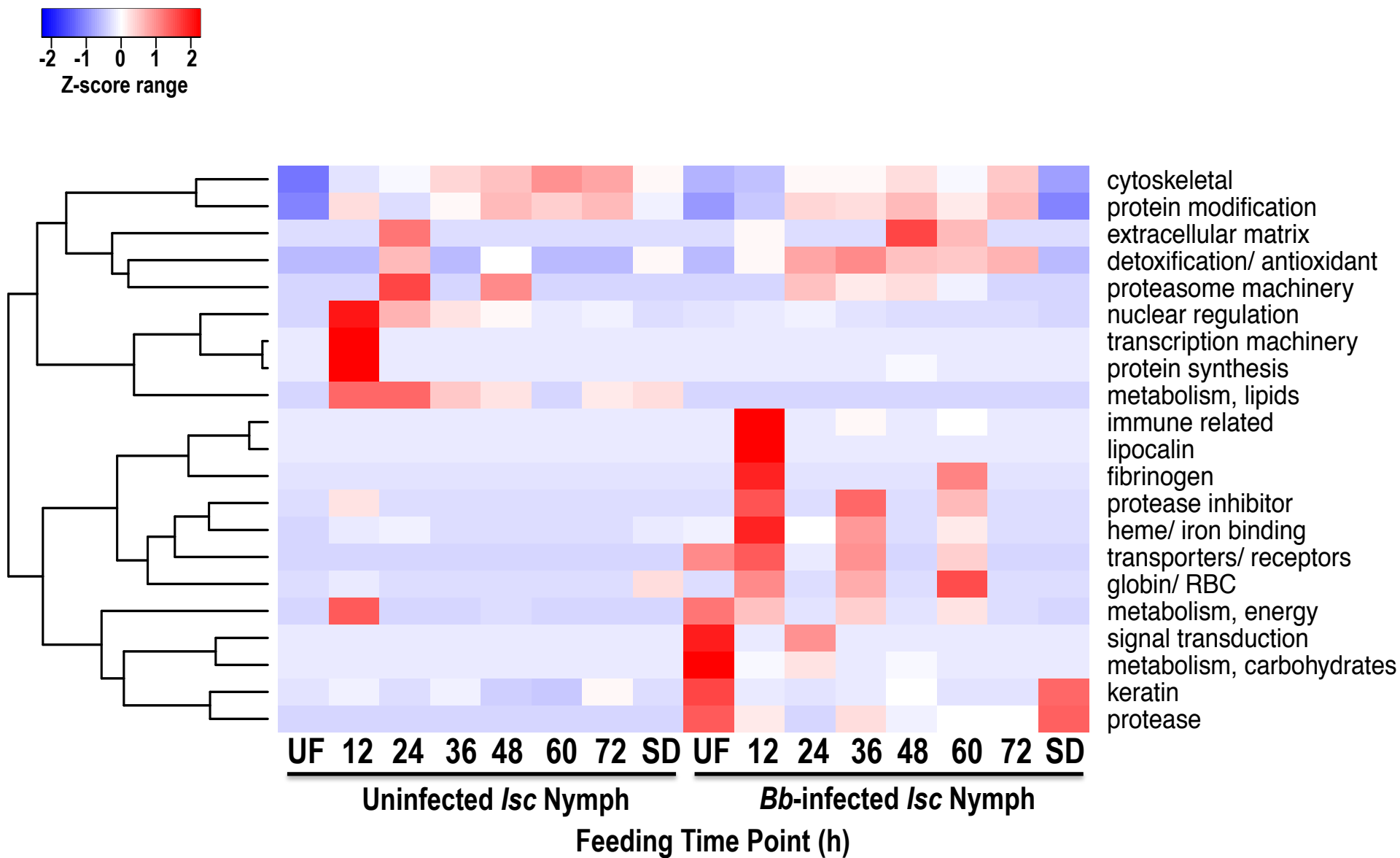

**Supplemental figure 6**

Supplement: Supplementary file 1 — Additional file 1: SF1. Non-invasive method of collecting saliva from Ixodes scapularis nymphs. Tick saliva collections were performed using a 10μl pipette tip set up. A modified 10μl pipette tip was used to affix the tick mouthpart in the solution and restrict the tick from escaping. Saliva collections from ticks were not included if leakage of fluid was detected around the protective cap. SF2. Antibody response to Borrelia burgdorferi antigens by ELISA and western blotting analyses. Total protein extracts from B. burgdorferi (1 or 3 μg) were coated per well for ELISA (A) or resolved by SDS-PAGE for western blotting (B) analyses using purified IgG (10μg/ ml) from pre-immune (PI), rabbit antibody (Ab) numbers 98, 25, 27, 50 and 51 from rabbits that were infested with uninfected nymphs and Ab numbers 97, 24, 26, 48, and 49 from rabbits that were infested with B. burgdorferi infected nymphs. For ELISA, the y-axis represents the A450 and x-axis represent the rabbit number. SF3. Profile of uninfected and Borrelia burgdorferi infected Ixodes scapularis nymph tick saliva proteins during feeding. Uninfected and B. burgdorferi infected I. scapularis nymph ticks that were unfed, partially fed for 12, 24, 36, 48, 60, and 72h, and replete-fed, were stimulated to salivate by injecting 2% pilocarpine into hemolymph. Saliva was electrophoresed on a 10-20% acrylamide gel and silver stained. Please note the molecular weight ladder from 10-250kDa. SF4. Secretion dynamics of all 747 proteins identified in uninfected and Borrelia burgdorferi infected Ixodes scapularis nymph tick saliva. Normalized spectral abundance factors (NSAF) values of all I. scapularis nymph tick saliva proteins identified in this study were normalized using the z-score statistics and then used to generate heat maps using heatmap2 function in gplots library using R as described in materials and methods. The red color represents high abundance to blue color indicating low abundance. SF5. Secretion dynamics of pro [file 12864_2021_7429_MOESM1_ESM.zip › SF6_ESM.pdf]
